# Direct Dehydrative Glycosylation Catalyzed by Diphenylammonium Triflate

**DOI:** 10.3390/molecules25051103

**Published:** 2020-03-02

**Authors:** Mei-Yuan Hsu, Sarah Lam, Chia-Hui Wu, Mei-Huei Lin, Su-Ching Lin, Cheng-Chung Wang

**Affiliations:** 1 Institute of Chemistry, Academia Sinica, Taipei 115, Taiwan; ja75822@gmail.com (M.-Y.H.); sarahlamyy@gmail.com (S.L.); ajhanne.chiahui@gmail.com (C.-H.W.); babylove333a@gmail.com (M.-H.L.); suching@gate.sinica.edu.tw (S.-C.L.); 2 Chemical Biology and Molecular Biophysics Program, Taiwan International Graduate Program (TIGP), Academia Sinica, Taipei 115, Taiwan; 3 Department of Chemistry, National Taiwan University, Taipei 106, Taiwan

**Keywords:** carbohydrates, dehydration, glycosylation, homogeneous catalysis microwave chemistry

## Abstract

Methods for direct dehydrative glycosylations of carbohydrate hemiacetals catalyzed by diphenylammonium triflate under microwave irradiation are described. Both armed and disarmed glycosyl-C1-hemiacetal donors were efficiently glycosylated in moderate to excellent yields without the need for any drying agents and stoichiometric additives. This method has been successfully applied to a solid-phase glycosylation.

## 1. Introduction

**Scheme 1:**
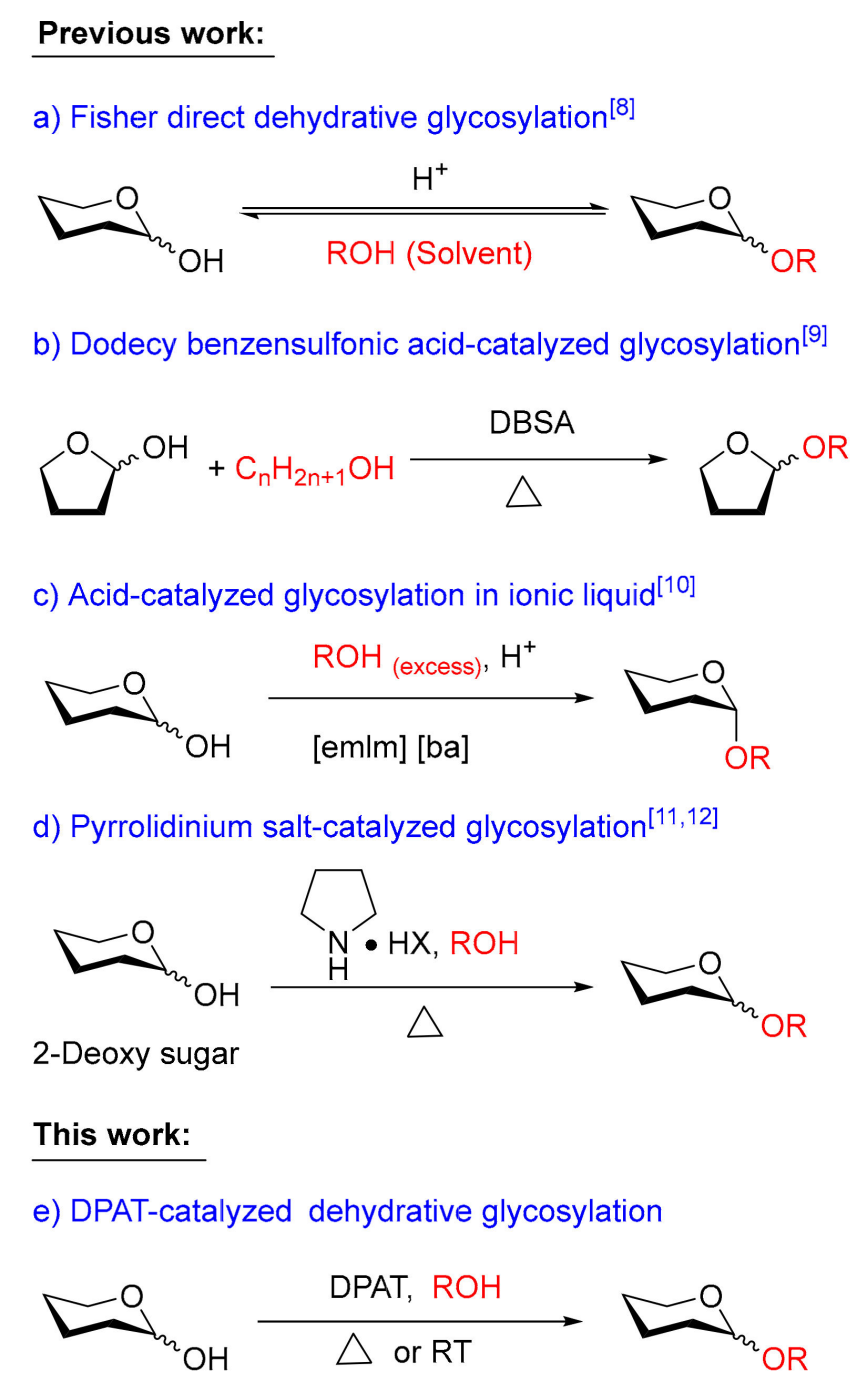
Direct dehydrative glycosylation.

Glycosylation is one of the most important reactions in oligosaccharide synthesis [1]. Though monosaccharides in hemiacetal form are commercially available or easily prepared, use of them as glycosyl donors often requires prior elaboration of the anomeric hydroxyl to a good leaving group [2,3,4,5,6,7]. In contrast, direct dehydrative glycosylation is an atom economic and environmentally friendly method because only water is generated as a byproduct. This approach has been utilized in classical Fischer glycosylation of unprotected sugars by using excess glycosyl acceptor and a stoichiometric amount of Brønsted acid as promoter [8]. More recently, direct dehydrative glycosylation has been achieved by using surfactant-type catalysts [9], ionic liquids as the reaction medium under acid catalysis [10] or pyrrolidinium salt as organocatalyst [11,12] (Scheme 1). However, these glycosylations are limited to the preparation of simple glycosides or 2-deoxy sugars, and application of this method to the synthesis of more complex oligosaccharides remains challenging.

Numerous metal-catalyzed condensation reactions have been reported in the literature [13,14]. The shift from metal to metal-free catalysis is the current trend for greener and more sustainable chemistry. Arylammonium triflates and bulky diarylammonium arenesulfonates were introduced by Tanabe [15,16] and Ishihara [17,18,19,20,21], respectively, to be effective catalysts for direct dehydrative esterification between carboxylic acids and alcohols in almost equimolar amounts. The local hydrophobic environment provided by the aryl substituents around the reaction center appears to enable condensation to proceed without the need to remove the water produced. We envisioned promoting dehydrative glycosylation in a similar manner. To date, there is just a single report describing an aggregated complex of a *N*,*N*-diarylammonium sulfate being used to catalyze dehydrative glycosylation of a reactive benzyl-protected ribose and 1-dodecanol in water [21]. Herein, we disclose a glycosylation reaction driven by water exclusion, which encompasses a microwave-assisted method for direct dehydrative glycosylations of both armed and disarmed saccharides by using diphenylammonium triflate (DPAT) as an efficient and green catalyst. No effort was made to remove or exclude water.

## 2. Results and Discussion

The process was initially applied to the reaction of 2,3,4,6-tetrabenzylglucose (**1**) with methanol in a 1:1 mixture of 1,2-dichloroethane (DCE) and toluene under microwave irradiation (Table 1). Using 10 mol% of the commercially available dimesityammonium pentafluorobenzenesulfonate (**3a**) [17] afforded methyl-*O*-glycoside **4a** as a mixture of α and β-anomers in 90% yield (Table 1, entry 2). Although the glycosylation could be promoted by conventional heating, microwave heating in general gave cleaner and more reliable results. The use of anhydrous solvents under inert atmosphere, so important in many previously reported glycosylations, was quite unnecessary. The workup procedure merely involved quenching with trimethylamine, followed by removal of solvent, and the desired glycosylation product was readily isolated by chromatography. The dehydrative glycosylation did not proceed without the catalyst under similar conditions (Table 1, entry 1).

Due to the relatively high cost of **3a**, we sought a cheaper alternative as catalyst. Diphenylammonium triflate (DPAT) (**3b**), which can be readily prepared by precipitation from a solution of equimolar diphenylamine and triflic acid in toluene [13], was next examined. We were gratified to find that methyl *O*-glycoside **4a** was formed in a similarly high yield under identical reaction conditions (entry 3). Addition of magnesium sulfate to scavenge water produced in the reaction did not improve the result (entry 4), indicating that water is kept outside the active reaction site by the phenyl groups of the catalyst so that the process is not sensitive to a small amount of water present in the reaction medium. Not unexpectedly, adding 5% H_2_O (v/v) to the reaction mixture inhibited glycosylation (entry 5); nonetheless, some product was formed, demonstrating that the local hydrophobic environment at the reaction center was not completely disrupted by the presence of a large amount of water. Based on the success of DPAT, a series of analogous ammonium salts was similarly prepared and screened for dehydrative glycosylation. In general, catalysts prepared from either dialkylamines (entries 6–7) or less acidic Brønsted acids, such as methanesulfonic acid (pKa (H_2_O) = –1.9, entry 8) [22], benzenesulfonic acid (pKa (H_2_O) = –2.8, entry 9) [17,18,19,20,21], or *p*-toluenesulfonic acid (pKa (H_2_O) = –2.1, entry 10) [23] were inactive. Only catalysts generated from Brønsted acids having acidity similar to that of triflic acid (pKa (H_2_O) = –14.7), such as perchloric acid (pKa (H_2_O) = –15.2) [24], gave comparable yields (entry 11). Note that using triflic acid alone led to a mixture of glycosylation products together with significant amounts of benzyl *O*-glycosides **4b**, arising from intermolecular benzyl group migration (entry 12).

Having identified DPAT as the most suitable catalyst for dehydrative glycosylation of **1** with methanol, the generality of the reaction with a panel of glycosyl acceptors was studied (Table 2). Under the same conditions, glycosylation of **1** with a range of primary (entries 1–2) and secondary (entries 3–4) alcohols proceeded smoothly to afford the corresponding glucosides in moderate to good yields. Less reactive acceptors including Cbz-protected amino acids **2f** and **2g** and primary monosaccharide **2h** also worked, although two equivalents of the acceptor were required for reasonable conversion (entries 5–7). No loss of the protecting groups on these acceptors was observed.

Next, the tolerance of the method for other protecting groups was explored (Table 3). Partial replacement of the electron-donating benzyl with electron-withdrawing acetyl or benzoyl groups had no effect on the reactivity of the donor toward glycosylation. For example, disaccharide **9h** was obtained from C6-acetyl-protected glycosyl donor **5** and monosaccharide acceptor **2h** in essentially the same yield as was **4h** from fully benzyl-protected **1** and **2h** (Table 3, entry 1 vs. Table 2, entry 7). Switching the anomeric protecting group to a thiol in the acceptor led to only a slightly diminished disaccharide yield (entry 2). Notably, the 1-thiol group, which was stable under the present dehydrative glycosylation, could serve as an orthogonal protection for an ensuing glycosylation. Triacylated and fully acylated donors **6**−**8** exhibited reactivity similar to that of **5** (entries 3–13). In addition, the 2-benzoyl group in **6** apparently engaged in a neighboring group participation that contributed to 100% β–selective glycosylations with the less reactive acceptors **2f**, **2h,** and **2i** (entries 5–7). The inactivity of secondary alcohol in monosaccharide acceptor can be advantageously exploited for regioselective glycosylation. For example, only the primary alcohol in 4,6-diol acceptor **2j** was reactive to undergo glycosylation to yield β-(1,6)-disaccharide **10j** as the sole product (entry 8). We note that **7** and **8** were previously reported to possess poor reactivity as glycosyl donors [25]. In some cases, slightly higher temperatures were required, but these systems also afforded gratifyingly decent glycosylation yields with simple alcohols (entries 9–11). The less reactive Cbz-protected serine **2f** afforded amino-sugar **11f** in moderate yield (entry 12). Under microwave irradiation at 80 °C in the presence of DPAT, the acetyl groups on **7** were partially cleaved, presumably making the donor more reactive towards glycosylation. In these cases, the crude reaction mixtures were subjected to re-acetylation prior to product isolation. For benzoyl-protected glucoside **8**, an even higher reaction temperature (100 °C) was necessary for glycosylation to proceed at reasonable rates (entries 13–15). In contrast to acetyl groups, benzoyl groups on **8** were more robust under our conditions and generally remained intact. Traces of 2-debenzoylated glycosylation products isolated in reactions with acceptors **2a** and **2d** suggested that the 2-acyl group was the most labile under high reaction temperatures and the prolonged reaction times that are required to activate highly disarmed donors such as **7** and **8** for glycosylation; this observation may account for the poorer stereoselectivies in glycosylation with **7** and **8** as donors.

Finally, the scope of the DPAT-catalyzed dehydrative glycosylation was examined using different sugars, including galactose **13**, mannose **14**, 2-deoxy sugars **17** and **18** (Table 4 and Table 5). The glycosylation products were obtained in reasonable yields up to 95% when using simple primary and secondary alcohols, serine derivative **2f** and monosaccharide **2k** as the glycosyl acceptors. Direct dehydative glycosylations with the more reactive 2-deoxy sugars **17** and **18** were accomplished at room temperature without microwave irradiation. Notably, the glycosylations favored the formation of α-anomers, and exclusive α-selectivity was realized with mannosyl donor **14** (Table 4, entries 5−8).

**Scheme 2:**
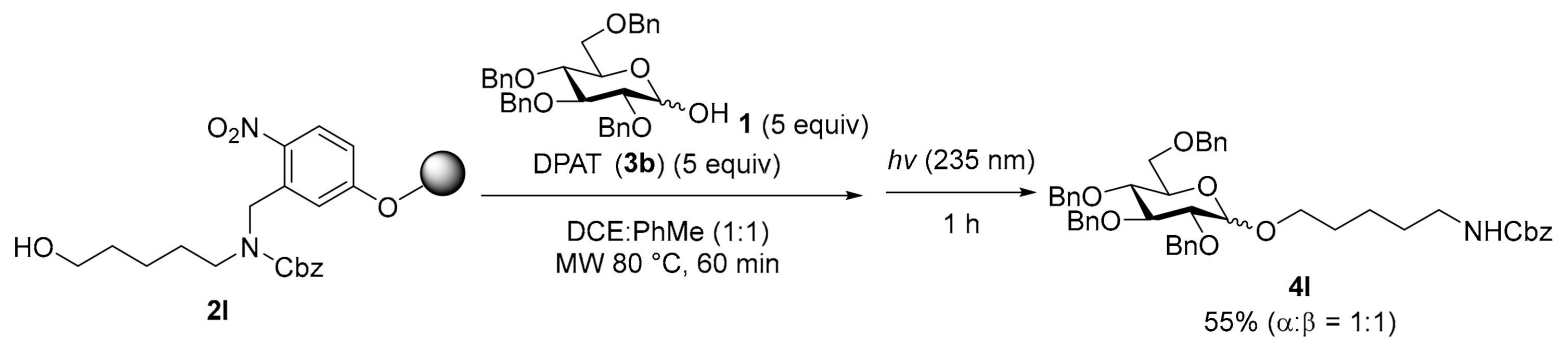
DPAT-promoted solid-phase dehydrative glycosylation.

Since the present dehydrative glycosylation was carried out under microwave heating, solid-phase glycosylation is likely to be performed using an ordinary peptide synthesizer. The applicability of the DPAT-promoted dehydrative glycosylation in solid-phase synthesis was then briefly investigated. To illustrate the feasibility of solid-phase dehydrative glycosylation, glycosyl acceptor **2l** immobilized on Merrifield resin with a photo-cleavable *o*-nitrobenzyl linker [26] was employed to react with **1** in the presence of DPAT under microwave irradiation at 80 °C for 1 h (Scheme 2). After a photo-induced cleavage from the solid support, the desired glycosylation product **4l** was obtained in 55% yield.

**Scheme 3:**
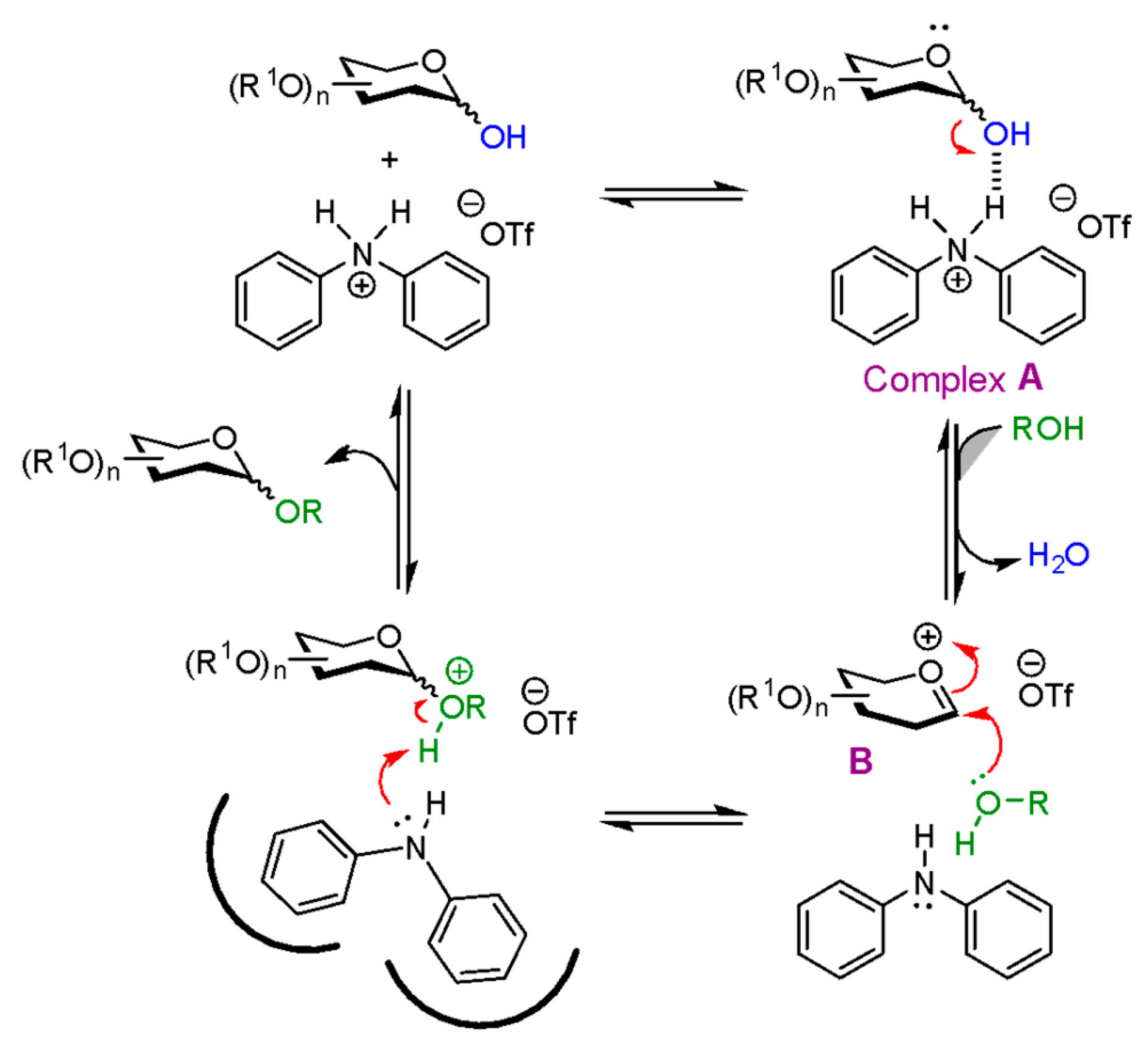
A plausible mechanism for dehydrative glycosylation.

To understand the mechanism of the DPAT-catalyzed dehydrative glycosylation, the reaction of 2-deoxyglucose **17** with isopropanol in dichloromethane-*d*_2_ at room temperature, similar to that shown in entry 1 of Table 5, was continuously monitored by ^1^H NMR spectroscopy (see SI). However, only the proton signals corresponding to the starting materials, catalyst, and product **19d** were observed over 2 h. Upon addition of DPAT, the proton signals of 2-doxyglucose **17** were broadened presumably due to their interactions through hydrogen bonding as depicted in complex **A** (see SI for more details). To know the variation of anomeric ratios of reactants and products over the course of reaction, DPAT-catalyzed dehydrative glycosylation reaction of methyl-*d_3_*-glucopyranose **21** and isopropanol (**2d**) was monitored by ^1^H NMR spectroscopy (see SI). Proton signals corresponding to anomeric mixtures **21α** and **21β** equilibrated to a ratio of 1:1 at the evaluated temperature and gradually diminished as the reaction progressed. Concurrently, proton signals corresponding to glycosides **22dα** and **22dβ** increased with a fixed equilibrium ratio (α:β = 1:0.7) (see Figures S4 and S5). Though the actual reaction mechanism awaits further investigation, Scheme 3 shows one plausible mechanism via oxacarbenium intermediate **B** formed by the elimination of a water molecule from the activated sugar. This oxacarbenium intermediate would be readily intercepted by an acceptor to furnish the corresponding glycosylation products. The water molecule would be expelled from the reaction center, and the hydrophobic environment created by the *N*-phenyl groups of the DPAT catalyst would prevent its re-entry, thereby driving the reaction to completion.

## 3. Conclusions

In conclusion, we report a direct dehydrative glycosylation reaction of carbohydrate hemiacetals catalyzed by diphenylammonium triflate under microwave irradiation. The hydrophobicity of diphenylammonium ions shields the reactive site from water to eliminate the formation of hydrolyzed products. This approach efficiently couples both armed and disarmed hemiacetal donors with a wide range of acceptors. No special precautions to exclude moisture or procedures to remove water generated during the course of the reaction are required. We have further applied this method to a solid-phase reaction using an acceptor immobilized on solid support. Initial mechanistic studies reveal that the glycosylation may involve a short-lived intermediate generated from DPAT-activation of the anomeric hydroxyl sugar. Detailed mechanistic studies and applications to automated solid-phase synthesis are currently underway.

## Figures and Tables

**Table 1:**
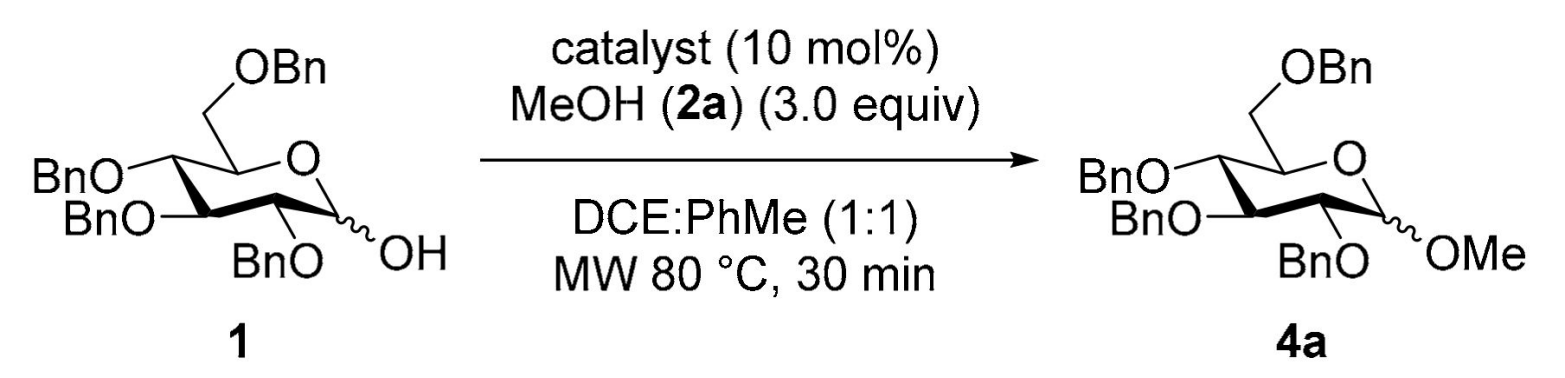
Initial catalyst screen for the glycosylation of **1** *^a^*.

Entry	Catalyst	Yield *^b^*	α:β *^c^*
1	-	NR *^d^*	-
2	[(Mes)_2_NH_2_][O_3_S(C_6_F_5_)] (**3a**)	90%	1:1
3	Ph_2_NH_2_OTf (DPAP) (**3b**)	90%	1:1
4 *^e^*	Ph_2_NH_2_OTf (DPAP) (**3b**)	83%	1:1
5 *^f^*	Ph_2_NH_2_OTf (DPAP) (**3b**)	9% *^g^*	1:1
6	Me_2_NH_2_OTf (**3c**)	NR	-
7	Bn_2_NH_2_OTf (**3d**)	NR	-
8	Ph_2_NH_2_OMs (**3e**)	5%*^g^*	ND *^h^*
9	Ph_2_NH_2_O_3_SPh (**3f**)	5%*^g^*	ND *^h^*
10	Ph_2_NH_2_OTs (**3g**)	5%*^g^*	ND *^h^*
11	Ph_2_NH_2_ClO_4_ (**3h**)	89%	1:1
12	TfOH	36% *^i^*	2:1

^a^ Reactions were performed with the following presentative procedure: To a solution of glycopyranose (0.2 mmol) in a 1:1 mixture of DCE and toluene (2.0 mL) in a flame-dried vessel or flask was added an acceptor (0.24–0.60 mmol) and diarylammonium salt (0.02 mmol) at room temperature under ambient atmosphere. The mixture was heated in a microwave reactor at target temperature. The progress of the reaction was monitored by TLC. After the reaction was complete, the reaction mixture was quenched by addition of triethylamine (0.03 mL, 0.2 mmol), concentrated under reduced pressure and the residue was purified by flash column chromatography on silica gel. ^b^ Isolated yield. ^c^ Determined by ^1^H NMR spectroscopy. ^d^ No reaction. ^e^ In the presence of 1.0 equiv. MgSO4. ^f^ In the presence of 5% (*v*/*v*) H_2_O. ^g^ 74%–87% **1** was recovered. ^h^ Not determined. ^i^ Along with 13% of 1,2,3,4,6-pentabenzylglucoside **4b**.

**Table 2:**
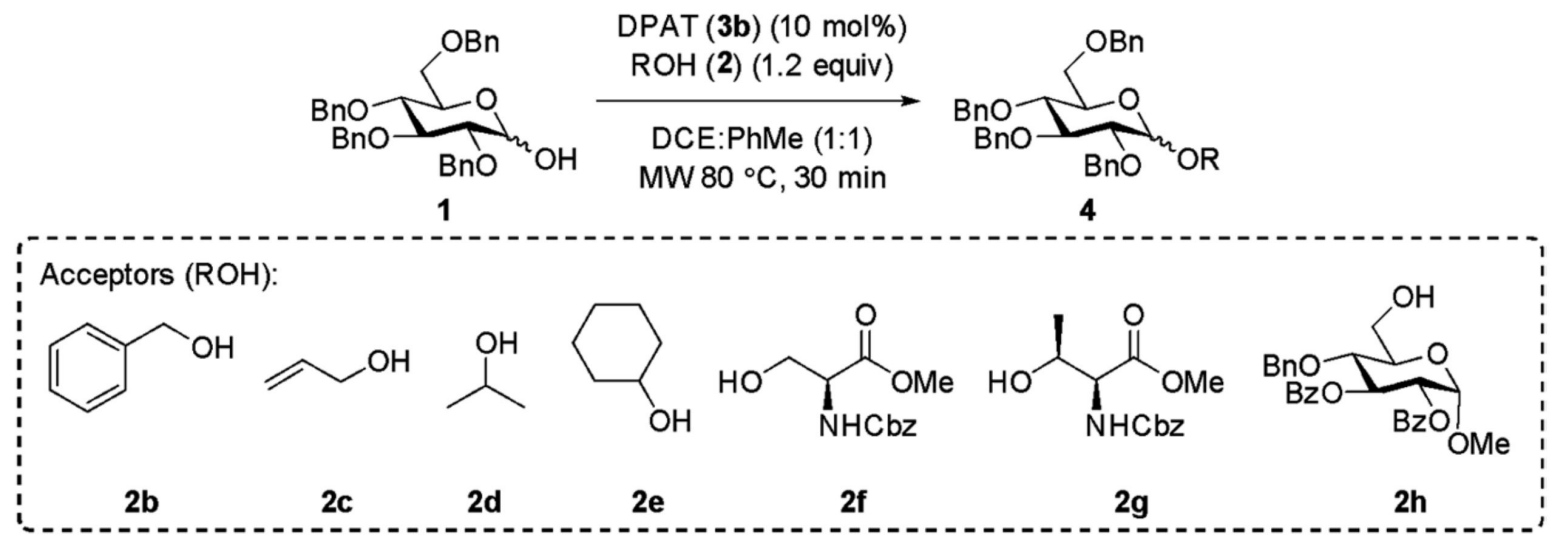
Acceptor scope with benzyl-protected glucose **1**.

Entry	Acceptor	Yield *^a^*	α:β *^b^*
1	benzyl alcohol (**2b**)	**4b** 75%	2:1
2	2-propenol (**2c**)	**4c** 80%	2:1
3	isopropanol (**2d**)	**4d** 74%	2:1
4	cyclohexanol (**2e**)	**4e** 76%	2:1
5 *^c^*	**2f**	**4f** 60%	2:1
6 *^c^*	**2g**	**4g** 48%	1:1
7 ^c,d,e^	**2h**	**4h** 64%	2:1

^a^ Isolated yield. ^b^ Determined by ^1^H NMR spectroscopy. ^c^ Using 2.0 equiv. of acceptor. ^d^ Reaction at 60 °C. ^e^ Reaction time = 60 min.

**Table 3:**
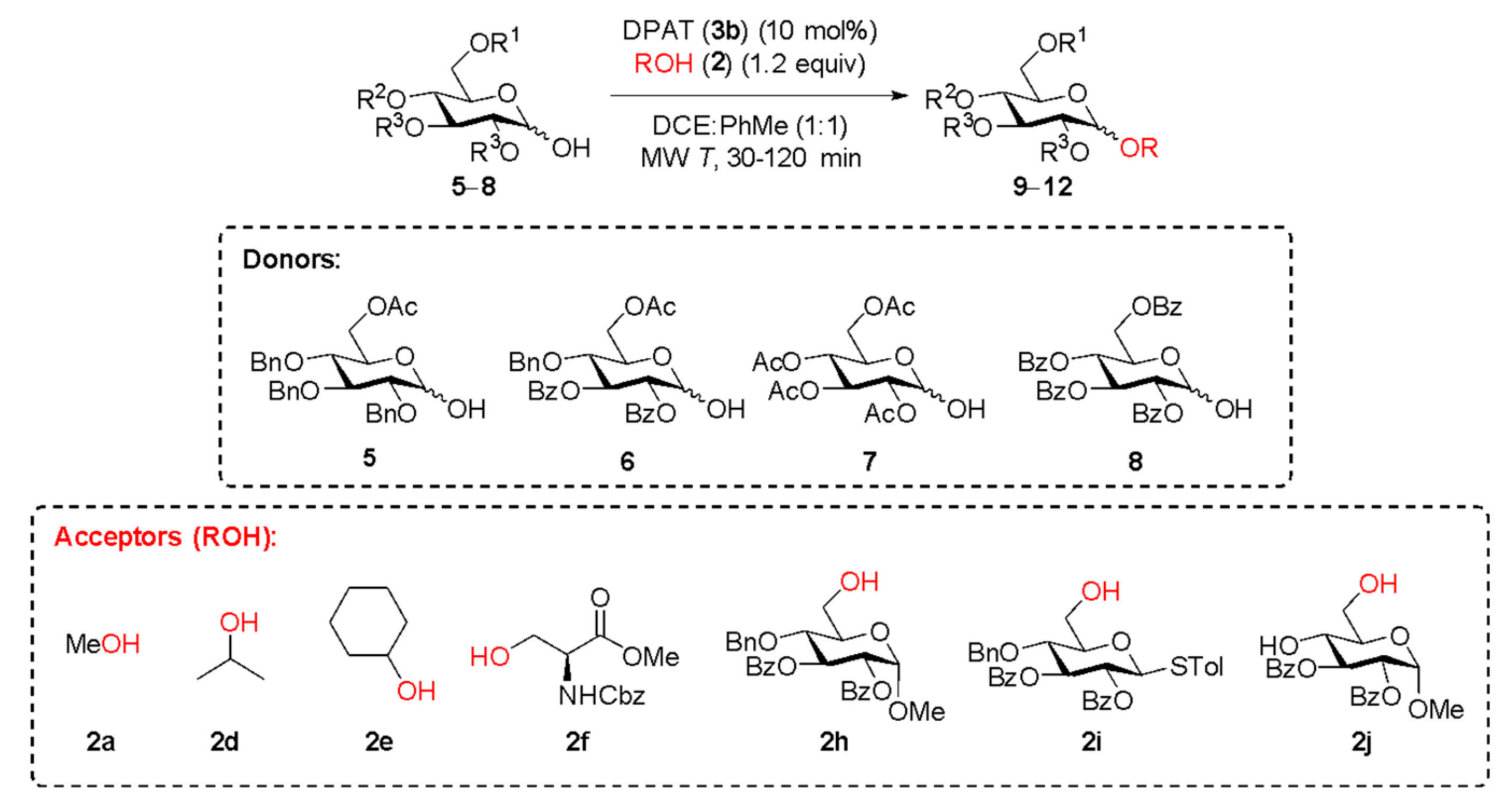
Reaction scope with various-protected glucoses.

Entry	Donor	Acceptor	*T* (°C)	Yield *^a^*	α:β *^b^*
1 *^c^*	**5**	**2h**	70	**9h** 68%	2:1
2 *^c^*	**5**	**2i**	70	**9i** 58%	3:1
3	**6**	**2d**	80	**10d** 71%	1:2
4	**6**	**2e**	80	**10e** 79%	1:2
5 *^c^*	**6**	**2f**	80	**10f** 60%	β-only
6 *^c^*	**6**	**2h**	70	**10h** 62%	β-only
7 *^c^*	**6**	**2i**	70	**10i** 56%	β-only
8 *^d^*	**6**	**2j**	80	10j 63%	β-only
9 *^e^*	**7**	**2a**	80	**11a** 58%	1:1
10 *^e^*	**7**	**2d**	80	**11d** 75%	2:1
11 *^e^*	**7**	**2e**	80	**11e** 62%	2:1
12 *^e^*	**7**	**2f**	100	**11f** 39%	1:1
13	**8**	**2a**	100	**12a** 64% *^f^*	1:2
14	**8**	**2d**	100	**12d** 75% *^f^*	3:1
15	**8**	**2e**	100	**12e** 71%	6:1

^a^ Isolated yield. ^b^ Determined by ^1^H NMR spectrocopy. ^c^ Using 2 equiv. of acceptor. ^d^ Using 1.8 equiv. of acceptor. ^e^ The crude product was treated with Ac_2_O and pyridine for 12–16 h prior to purification. ^f^~5% of 2-debenzoylated glycosylation product isolated.

**Table 4:**
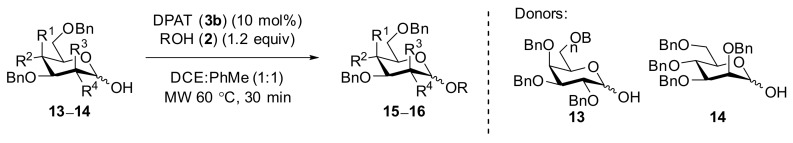
Reaction scope of diphenylammonium triflate (DPAT)-catalyzed dehydrative glycosylation of galactose and mannose.

Entry	Donor	Acceptor	Yield *^a^*	α:β *^b^*
1 *^c^*	**13**	**2a**	**15a** 95%	2:1
2 ^d,e^	**13**	**2d**	**15d** 82%	2:1
3	**13**	**2e**	**15e** 59%	2:1
4	**13**	**2f**	**15f** 56%	2:1
5 *^c^*	**14**	**2a**	**16a** 90%	α only
6 ^d,e^	**14**	**2d**	**16d** 82%	α only
7	**14**	**2e**	**16e** 73%	α only
8	**14**	**2f**	**16f** 62%	α only

^a^Isolated yield. ^b^Determined by ^1^H NMR spectroscopy. ^c^Using 3 equiv. of acceptor. ^d^Using 2.4 equiv. of acceptor. ^e^Reaction time = 60 min.

**Table 5:**
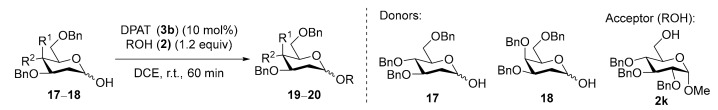
Reaction scope of DPAT-catalyzed dehydrative glycosylation of 2-deoxyglycoses.

Entry	Donor	Acceptor	Yield *^a^*	α:β *^b^*
1	**17**	**2d**	**19d** 68%	3:1
2	**17**	**2e**	**19e** 60%	3:1
3	**17**	**2f**	**19f** 52%	5:1
4	**17**	**2k**	**19k** 51%	4:1
5	**18**	**2d**	**20d** 70%	6:1
6	**18**	**2e**	**20e** 73%	6:1
7	**18**	**2f**	**20f** 68%	10:1
8	**18**	**2k**	**20k** 51%	6:1

^a^ Isolated yield. ^b^ Determined by ^1^H NMR spectroscopy.

## Supplementary Materials

The following are available online at https://www.mdpi.com/1420-3049/25/5/1103/s1, Figure S1: 1H NMR spectra of reaction of **17** and **2d** in the presence the of 10 mol% of DPAT (**3b**) in CD_2_Cl_2_ at the ambient temperature for a) 10 min; b) 20 min; c) 30 min: d) 40 min; e) 50 min; f) 60 min; g) 120 min. Figure S2: 1H NMR spectra in CD_2_Cl_2_ of a) **17**; b) 10:1 of mixture of **17** and DPAT **(3b**) c) 10:1 of mixture of **17** and [(Mes)_2_NH_2_][O_3_S(C_6_F_5_)] (**3a**). Figure S3: 1H NMR spectra of DPAT-catalyzed reaction of **21** and **2d** in toluene-*d*_8_ at a) ambient temperature for 1 h; b) 60 ^o^C for 30 min; c) 70 ^o^C for 20 min; d) 80 ^o^C for 10 min; e) 80 ^o^C for 30 min; and f) 80 ^o^C for 50 min. Figure S4: The normalized plots of methyl-*d_3_*-glucopyranose **21**. Black line indicates **21**, red line indicates **21****α**, and blue line indicates **21β**. Figure S5: The normalized plots of glucoside **22d**. Black line indicates **22**, red line indicates **α-22d**, and blue line indicates **β-22d**. Figure S6: The water-repelling study in 1,2-dichloroethane of a) H_2_O; b) 10:1 of mixture of H_2_O and Ph_2_NH_2_; c) 10:1 of mixture of H_2_O and TfOH; d) 10:1:1 of mixture of H_2_O, TfOH, and succinimide; e) 10:1 of mixture of H_2_O and DPAT (**3b**); f) 10:1 of mixture of H_2_O and [(Mes)_2_NH_2_][O_3_S(C_6_F_5_)] (**3a**).
