# Supplementary material for: Direct Dehydrative Glycosylation Catalyzed by Diphenylammonium Triflate
Source: Molecules. 2020 Mar 2;25(5):1103. doi: 10.3390/molecules25051103 (PMC7179217; doi:10.3390/molecules25051103)
Supplement: Supplementary file 1 [file molecules-25-01103-s001.pdf]

Supporting Information

Direct Dehydrative Glycosylation Catalyzed by Diphenylammonium Triflate

Mei-Yuan Hsu, [1,2,3†] Sarah Lam,[1†] Chia-Hui Wu,[1,2,3] Mei-Huei Lin,[1] Su-Ching Lin,[1] Cheng-Chung Wang*[1,2]

[1] Institute of Chemistry, Academia Sinica, Taipei 115, Taiwan
[2] Chemical Biology and Molecular Biophysics Program, Taiwan International Graduate Program (TIGP), Academia Sinica, Taipei 115, Taiwan
[3] Department of Chemistry, National Taiwan University, Taipei 106, Taiwan
[†] Both authors contributed equally to this work.

Table of contents

General experimental 2

General procedure A: Preparation of diarylammonium catalyst 2

General procedure B: Microwave-assisted DPAT-catalyzed dehydrative glycosylation 3

General procedure C: Microwave-assisted DPAT-catalyzed dehydrative glycosylation of 2,3,4,6-tetra-*O*-acetyl-glucopyranose **7** 3

General procedure D: DPAT-catalyzed dehydrative glycosylation of 2-deoxyglycopyranoses (Table 5) 3

Preparation of glycosyl donors **6** 4

Dehydrative glycosylation reactions of hemiacetal donors 5

NMR studies of glycosylation mechanism (Figure S1-S5) 40

Water-repelling study (Figure S6) 44

References 45

^{1}H and ^{13}C NMR spectra 47

General Experimental. Microwave-assisted reactions were performed using the Discover SP System (CEM). All reagents obtained from commercial sources were utilized without purification unless otherwise specified. Reactions were monitored by thin layer chromatography (TLC) using pre-coated glass plates of Silica Gel 60 F254 (0.25 mm, E. Merck). Components were visualized by illumination with short-wavelength ultra-violet light and/or staining by spraying with a solution of $Ce(NH_4)_2(NO_3)_6$, $(NH_4)_6Mo_7O_{24}$ and H_2SO_4 in water and subsequent heating on a hot plate. Flash column chromatography was carried out with Silica Gel 60 (230-400 mesh, E. Merk). Optical rotations were measured on a JASCO P-2000 polarimeter. ^1H and ^{13}C NMR spectra were recorded in deuteriochloroform ($CDCl_3$) at ambient temperature on with Bruker DRX 500 MHz, AV500, AV400 and AVIII 400 MHz spectrometer. All spectra were calibrated at δ 7.24 or δ 0.00 ppm for ^1H spectra (residual $CHCl_3$ or TMS respectively), and 77.23 ppm for ^{13}C spectra. Splitting patterns were designated as follows: s = singlet, d = doublet, t = triplet, q = quartet, m = multiplet, br = broad; *J* = coupling constant value in hertz (Hz). Mass spectra were analyzed by a Waters Premier XE mass spectrometer with electrospray ionization (ESI) mode.

Glycosyl donors **1**[1], **5**[2], **7**[3], **8**[4], **13**[5], **14**[6], **17**[7], **18**[8] and glycosyl acceptors **2h**[9], **2i**[10], **2j**[11], **2k**[12], **2l**[13] were prepared according to literature procedures. The other acceptors were commercially available and directly used without purification.

General procedure A: Preparation of diarylammonium catalyst. To a solution of amine derivative (1.0 mmol) in toluene (2.0 mL) was dropwise added Lewis acid (1.1 mmol) at room temperature under ambient atmosphere, and stirried

for 30 min. The mixture was fitered and washed with hexane to afford diarylammonium salt as a white solid.

General procedure B: Microwave-assisted DPAT-catalyzed dehydrative glycosylation. To a solution of glycopyranose (0.2 mmol) in a 1:1 mixture of DCE and toluene (2.0 mL) was added acceptor (0.24–0.60 mmol) and diarylammonium salt (0.02 mmol) in a flame-dried vessel or flask at room temperature under ambient atmosphere. The mixture was heated in a microwave reactor at target temperature. The progress of the reaction was monitored by TLC. After the reaction was complete, the reaction mixture was quenched by addition of triethylamine (0.03 mL, 0.2 mmol), concentrated under reduced pressure and the residue was purified by flash column chromatography on silica gel.

General procedure C: Microwave-assisted DPAT-catalyzed dehydrative glycosylation of 2,3,4,6-tetra-*O*-acetyl-glucopyranose 7. To a solution of glucopyranose **7** (0.2 mmol) in a 1:1 mixture of DCE and toluene (2.0 mL) was added acceptor (0.24–0.60 mmol) and diarylammonium salt (0.02 mmol) in a flame-dried vessel or flask at room temperature under ambient atmosphere. The mixture was heated in a microwave reactor at 80 °C. The progress of the reaction was monitored by TLC. After the reaction was complete, the reaction mixture was quenched by addition of triethylamine (0.03 mL, 0.2 mmol), concentrated under reduced pressure. Pyridine (1.0 mL) and Ac_2O (1.0 mL) was added. The resulting mixture was stirred at room temperature for 12–16 h and then concentrated under reduced pressure. The residue was purified by flash column chromatography on silica gel.

General procedure D: DPAT-catalyzed dehydrative glycosylation of 2-deoxyglycopyranoses (Table 5). To a solution of 2-deoxyglycopyranose (0.2

mmol) in DCE (2.0 mL) was added acceptor (0.24–0.40 mmol) and DPAT (0.02 mmol) in a flame dried flask at room temperature under ambient atmosphere. The progress of the reaction was monitored by TLC. After the reaction is complete, the reaction mixture was quenched by addition of triethylamine (0.03 mL, 0.2 mmol), concentrated under reduced pressure and the residue was purified by flash column chromatography on silica gel.

Preparation of glycosyl donors 6

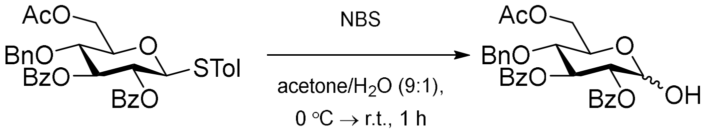

To a solution of thioglucoside **S1**[14] (6.3 g, 10 mmol) in acetone/H_2O (9:1, 50 mL) was added *N*-bromosuccinimide (2.7 g, 15 mmol) at 0 °C under ambient atmosphere. The mixture was gradually warm up to room temperature and stirred for 1 h. The mixture was neutralized with aqueous $NaHCO_3$ (50 mL × 2). The organic layer was sepearated, and the aqueous layer was extracted with EtOAc (50 mL × 2). The combined organic phase was washed with brine (50 mL × 2), dried over anhydrous $MgSO_4$, filtered, and then concentrated under reduced pressure. The crude product was purified by flash column chromatography using 0/1 to 1/2 EtOAc/hexane to afford **6** (3.1 g, 61%). **2,3-Di-*O*-benzoyl-4-*O*-benzyl-6-*O*-acetyl-1-D-glucopyranose (6)**: Colorless oil; $[\alpha]^{26}_{D}$ +99.2 (*c* 2.3, CH_2Cl_2); IR (CH_2Cl_2) ν 1720, 1261, 1094, 1042, 1027, 732 cm^{-1}; ^1H NMR (400 MHz, $CDCl_3$) δ 7.99–7.12 (m, 30H, Ar*H*), 6.10 (t, *J* = 9.6 Hz, 1H, 3-Hα), 5.78 (t, *J* = 9.2 Hz, 1H, H-3β), 5.60 (d, *J* = 3.6 Hz, 1H, H-1α), 5.19 (dd, *J* = 9.2, 7.2 Hz, 1H, H-2β), 5.16 (dd, *J* = 9.2, 3.6 Hz, 1H, H-2α), 4.91 (t, *J* = 7.2 Hz, 1H, H-1β), 4.62–4.49 (d, *J* = 10.8 Hz, 4H, ArC*H*), 4.43–4.39 (m, 2H, H-6α, H-6β), 4.32–4.24

(m, 3H, H-6α, H-6β, H-5α), 3.89–3.75 (m, 3H, H-4β, H-4α, H-5β), 2.07 (m, 6H, CH_3) ppm; ^{13}C NMR (100 MHz, $CDCl_3$) δ 170.8 (C), 166.9 (C), 166.0 (C), 165.6 (C), 137.0 (C), 133.5 (CH), 129.9 (CH), 129.2 (CH), 129.0 (CH), 128.8 (CH), 128.6 (CH), 128.5 (CH), 128.4(CH), 128.3 (CH), 128.2 (CH), 128.1 (CH), 128.0 (CH), 95.7 (C-1α), 90.3 (C-1β), 75.8 (CH), 74.7 (CH_2), 74.3 (CH_2), 73.3 (CH), 72.3 (CH), 68.6 (CH), 62.7 (CH_2), 20.8 (CH) ppm; HRMS (ESI) *m*/*z* calcd for $C_{29}H_{28}O_9Na$ ([M + Na]$^+$) 543.1631, found 543.1635.

<u>**Dehydrative glycosylation reactions of glycopyranoses**</u>

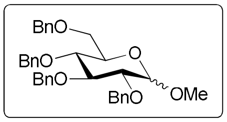

Methyl 2,3,4,6-tetra-*O*-benzyl-D-glucopyranoside (4a).

According to the general procedure B for the glycosylation, glucopyranose **1** (108.0 mg, 0.1998 mmol) in 1:1mixture of DCE and toluene (2.0 mL) was subjected to reaction with MeOH (**2a**) (18 μL, 0.44 mmol) and DPAT **3b** (6.5 mg, 0.020 mmol) under microwave irradiation at 80 °C for 30 min. The crude product was purified by flash column chromatography to afford compound **4a** (99.8 mg, 90%, α:β = 1:1). The spectroscopic data of **4a** was in agreement with those previously reported in the literature.[15]

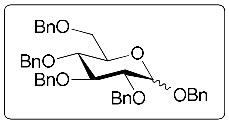

Benzyl 2,3,4,6-tetra-*O*-benzyl-D-glucopyranoside (4b).

According to the general procedure B for the glycosylation, glucopyranose **1** (108.0 mg, 0.1998 mmol) in 1:1mixture of DCE and toluene (2.0 mL) was subjected to reaction with BnOH (**2b**) (25 μL, 0.24 mmol) and DPAT **3b** (6.5 mg, 0.020 mmol) under microwave irradiation at 80 °C for 30 min. The crude product was purified by flash column chromatography to afford compound **4b**

(94.6 mg, 75%, α:β = 2:1). The spectroscopic data of **4b** was in agreement with those previously reported in the literature. [2]

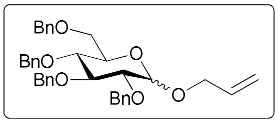

Allyl 2,3,4,6-tetra-*O*-benzyl-D-glucopyranoside (4c).

According to the general procedure B for the glycosylation, glucopyranose **1** (108.0 mg, 0.1998 mmol) in 1:1mixture of DCE and toluene (2.0 mL) was subjected to reaction with allyl alcohol (**2c**) (16 μL, 0.24 mmol) and DPAT **3b** (6.5 mg, 0.020 mmol) under microwave irradiation at 80 °C for 30 min. The crude product was purified by flash column chromatography to afford compound **4c** (93.2 mg, 80%, α:β = 2:1). The spectroscopic data of **4c** was in agreement with those previously reported in the literature.[16,17]

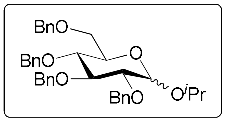

Isoproropyl 2,3,4,6-tetra-*O*-benzyl-D-glucopyranoside (4d).

According to the general procedure B for the glycosylation, glucopyranose **1** (108.0 mg, 0.1998 mmol) in 1:1mixture of DCE and toluene (2.0 mL) was subjected to reaction with isopropanol (**2d**) (18 μL, 0.24 mmol) and DPAT **3b** (6.5 mg, 0.020 mmol) under microwave irradiation at 80 °C for 30 min. The crude product was purified by flash column chromatography to afford compound **4d** (85.9 mg, 74%, α:β = 2:1). The spectroscopic data of **4d** was in agreement with those previously reported in the literature.[18]

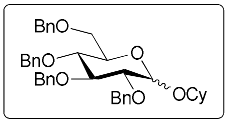

Cyclohexyl 2,3,4,6-tetra-*O*-benzyl-D-glucopyranoside (4e).

According to the general procedure B for the glycosylation, glucopyranose **1**

(108.0 mg, 0.1998 mmol) in 1:1mixture of DCE and toluene (2.0 mL) was subjected to reaction with cyclohexenol (**2e**) (25 μL, 0.24 mmol) and DPAT **3b** (6.5 mg, 0.020 mmol) under microwave irradiation at 80 °C for 30 min. The crude product was purified by flash column chromatography to afford compound **4e** (94.7 mg, 76%, *α*:β = 2:1). The spectroscopic data of **4e** was in agreement with those previously reported in the literature.[18]

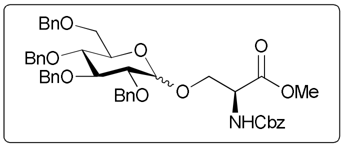

***O*-[2,3,4,6-Tetra-*O*-benzyl-D-glucopyranosyl]-*N*-carbobenzyloxy-L-serine methyl ester (4f).**

According to the general procedure B for the glycosylation, glucopyranose **1** (108.0 mg, 0.1998 mmol) in 1:1mixture of DCE and toluene (2.0 mL) was subjected to reaction with *N*-Cbz-L-serine methyl ester (**2f**) (101.0 mg, 0.4293 mmol) and DPAT **3b** (6.5 mg, 0.020 mmol) under microwave irradiation at 80 °C for 30 min. The crude product was purified by flash column chromatography to afford compound **4f** (93.1 mg, 60%, *α*:β = 2:1). The spectroscopic data of **4f** was in agreement with those previously reported in the literature.[19]

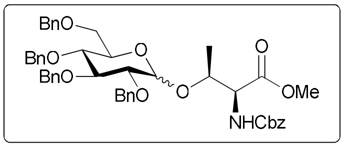

***O*-[2,3,4,6-Tetra-*O*-benzyl-D-glucopyranosyl]-*N*-carbobenzyloxy-L-threonine methyl ester (4g).**

According to the general procedure B for the glycosylation, glucopyranose **1** (108.0 mg, 0.1998 mmol) in 1:1mixture of DCE and toluene (2.0 mL) was

subjected to reaction with *N*-Cbz-L-threonine methyl ester (**2g**) (107.0 mg, 0.4003 mmol) and DPAT **3b** (6.5 mg, 0.020 mmol) under microwave irradiation at 80 °C for 30 min. The crude product was purified by flash column chromatography to afford compound **4g** (75.8 mg, 48%, α:β = 1:1). The spectroscopic data of **4g** was in agreement with those previously reported in the literature.[19]

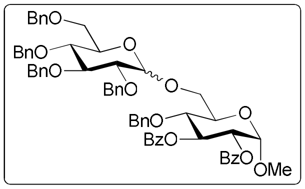

Methyl 2,3-di-*O*-benzoyl-4-*O*-benzyl-6-*O*-(2,3,4,6-tetra-*O*-benzyl-D-glucopyranosyl)-α-D-glucopyranoside (4h).

According to the general procedure B for the glycosylation, glucopyranose **1** (108.0 mg, 0.1998 mmol) in 1:1mixture of DCE and toluene (2.0 mL) was subjected to reaction with monosaccharide acceptor (**2h**) (196.0 mg, 0.3980 mmol) and DPAT **3b** (6.5 mg, 0.020 mmol) under microwave irradiation at 60 °C for 60 min. The crude product was purified by flash column chromatography to afford compound **4h** (129.9 mg, 64%, α:β = 2:1).

4h: Colorless oil; $[\alpha]^{26}_{D}$ +83.7 (*c* 2.70, CH_2Cl_2); IR (CH_2Cl_2) ν 3366, 1725, 1496, 1095, 1068, 1015, 1095, 734, 696 cm^{-1}; ^{1}H NMR (400 MHz, $CDCl_3$) δ 8.0 (m, 6H, Ar*H*), 7.93 (d, *J* = 7.6 Hz, 2H, Ar*H*), 7.88 (d, *J* = 7.6 Hz, 4H, Ar*H*), 7.50–7.10 (m, 92H, Ar*H*), 6.06 (m, 3H), 5.21 (m, 2H), 5.19 (m, 4H), 5.13 (d, *J* = 7.2 Hz, 1H), 5.08 (m, 2H), 5.05 (m, 2H), 4.98 (d, *J* = 7.2 Hz, 1H), 4.89 (m, 3H), 4.88 (m, 4H), 4.85 (m, 4H), 4.63 (m, 4H), 4.62 (m, 4H), 4.53 (m, 2H), 4.52 (m, 4H), 4.45 (d, *J* = 7.6 Hz, 1H), 4.29 (dd, , *J* = 11.2, 3.6 Hz, 1H), 4.93 (m, 3H), 4.02 (m, 4H), 4.01 (d, *J* = 2.8 Hz, 1H), 3.98 (m, 2H), 3.89 (m, 4H), 3.83, (dd, *J* = 11.2, 3.6 Hz, 1H), 3.77 (m, 3H),

3.75 (m, 3H), 3.73 (m, 3H), 3.67 (m, 4H), 3.60 (t, *J* = 9.6 Hz, 1H), 3.49 (m, 1H), 3.40 (s, 6H), 3.39 (s, 3H) ppm; ^{13}C NMR (100 MHz, $CDCl_3$) δ 166.2 (C), 166.1 (C), 165.8 (C), 165.7 (C), 139.0 (C), 138.7 (C), 138.6 (C), 138.2 (C), 137.8 (C), 133.1 (CH), 129.9 (CH), 129.8 (CH), 129.4 (CH), 128.6 (CH), 128.5 (CH), 128.4 (CH), 128.2 (CH), 128.1 (CH), 128.0 (CH), 127.9 (CH), 127.8 (CH), 127.7 (CH), 104.1 (CH), 97.4 (CH), 97.1 (CH), 97.0 (CH), 84.9 (CH), 82.4 (CH), 81.9 (CH), 80.4 (CH), 78.1 (CH), 77.5 (CH), 76.1 (CH), 75.9 (CH_2), 75.7 (CH), 75.3 (CH_2), 75.2 (CH_2), 75.0 (CH), 74.8 (CH_2), 74.7 (CH_2), 74.6 (CH_2), 72.8 (CH), 72.5 (CH), 70.8 (CH), 70.6 (CH), 69.2 (CH_2), 68.7 (CH_2), 68.5 (CH_2), 65.2 (CH_2), 60.5 (CH_2), 55.5 (CH_3), 21.0 (CH_3), 14.3 (CH_3) ppm; HRMS (ESI) *m*/*z* calcd for $C_{62}H_{62}O_{13}Na$ ($[M + Na]^+$) 1037.4088, found 1037.4082.

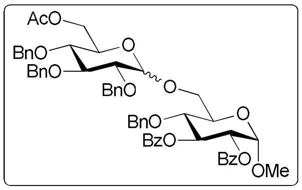

Methyl 2,3-di-*O*-benzoyl-4-*O*-benzyl-6-*O*-(2,3,4-tri-*O*-benzyl-6-*O*-acetyl-D-glucopyranosyl)-*α*-D-glucopyranoside (9h).

According to the general procedure B for the glycosylation, glucopyranose **5** (98.5 mg, 0.200 mmol) in 1:1mixture of DCE and toluene (2.0 mL) was subjected to reaction with monosaccharide acceptor **2h** (196.0 mg, 0.3980 mmol) and DPAT **3b** (6.5 mg, 0.020 mmol) under microwave irradiation at 70 °C for 60 min. The crude product was purified by flash column chromatography to afford compound **9h** (131.3 mg, 68%, *α*:β = 2:1).

***α*-9h**: Colorless oil; $[\alpha]^{26}_{D}$ +69.8 (*c* 2.63, CH_2Cl_2); IR (CH_2Cl_2) ν 1724, 1274, 1092, 1068, 1026, 739, 698 cm^{-1}; ^{1}H NMR (400 MHz, $CDCl_3$) δ 7.95 (d, *J* = 7.6 Hz, 2H, Ar*H*), 7.86 (d, *J* = 7.6 Hz, 2H, Ar*H*), 7.47 (m, 4H, Ar*H*), 7.37–7.26 (m, 17H, Ar*H*),

7.17 (m, 2H), 7.10 (m, 3H), 5.99 (dd, *J* = 9.6, 9.2 Hz, 1H, H-3′), 5.15 (d, *J* = 3.6 Hz, 1H, H-1), 5.14 (d, *J* = 3.6 Hz, 1H, H-1′), 5.06 (d, *J* = 10.8 Hz, 1H, ArC*H*), 5.01 (dd, *J* =9.6, 3.6, Hz, 1H, H-2′), 4.90 (d, *J* = 10.8 Hz, 1H, ArC*H*), 4.86 (d, *J* = 10.8 Hz, 1H, ArC*H*), 4.83 (d, *J* = 12.0 Hz, 1H, ArC*H*), 4.81 (d, *J* = 12.0 Hz, 1H, ArC*H*), 4.63 (s, 2H, ArC*H*), 4.58 (d, *J* = 10.8 Hz, 1H, ArC*H*), 4.26 (m, 2H, H-6′), 4.07 (t, *J* = 9.2 Hz, 1H, H-4′), 4.01 (m, 2H, H-6, H-5), 3.93 (m, 2H, H-6, H-5′), 3.87 (t, *J* = 9.2 Hz, 1H, H-3), 3.63 (dd, *J* =9.2, 3.6, Hz, 1H, H-2), 3.53 (t, *J* = 9.2 Hz, 1H, H-4), 3.37 (s, 3H), 2.01 (s, 3H) ppm; ^{13}C NMR (100 MHz, $CDCl_3$) δ 170.7 (C), 165.9 (C), 165.6 (C), 138.6 (C), 138.6 (C), 138.2 (C), 137.9 (C), 133.2 (CH), 132.9 (CH), 129.9 (CH), 129.7 (CH), 129.2 (CH), 129.0 (CH), 128.5 (CH), 128.4 (CH), 128.3 (CH), 128.2 (CH), 128.1 (CH), 128.0 (CH), 127.8 (CH), 127.7 (CH), 127.6 (CH), 97.0 (CH),96.8 (CH), 81.7 (CH), 80.2 (CH), 77.3 (CH), 75.8 (CH), 75.6 (CH_2), 75.0 (CH_2), 74.5 (CH_2), 72.7 (CH_2), 72.5 (CH), 70.6 (CH), 68.9 (CH), 65.1 (CH_2), 63.0 (CH_2), 55.3 (CH_3), 20.8 (CH_3) ppm; HRMS (ESI) *m*/*z* calcd for $C_{57}H_{58}O_{14}Na$ ([M + Na]$^+$) 989.3724, found 989.3715.

β-9h: Colorless oil; $[\alpha]^{29}_{D}$ +55.7 (*c* 2.25, CH_2Cl_2); IR (CH_2Cl_2) ν 1726, 1602, 1453, 1274, 1091, 1069, 1027, 737, 699 cm^{-1}; ^{1}H NMR (400 MHz, $CDCl_3$) δ 7.97 (d, *J* = 8.4 Hz, 2H, ArC*H*), 7.91 (d, *J* = 8.4 Hz, 2H, Ar*H*), 7.49 (m, Ar*H*), 7.41–7.26 (m, 18H, Ar*H*), 7.20 (m, 4H, Ar*H*), 7.14 (m, 2H, Ar*H*), 6.04 (t, *J* =9.6 Hz, 1H, H-3′), 5.15 (m, 2H, H-2′, H-1′), 5.11 (d, *J* = 10.8 Hz, 1H, ArC*H*), 4.99 (d, *J* =10.8 Hz, 1H, ArC*H*), 4.89 (d, *J* =10.8 Hz, 1H, ArC*H*), 4.87 (d, *J* =10.8 Hz, 1H, ArC*H*), 4.84 (d, *J* =10.8 Hz, 1H, ArC*H*), 4.59 (d, *J* =10.8 Hz, 1H, ArC*H*)), 4.48 (s, 2H, ArC*H*), 4.41 (d, *J* =8.0 Hz, 1H, H-1), 4.37 (d, *J* =10.8, 1.6 Hz, 1H, H-6), 4.22 (m, 2H, H-6, H-6′), 4.06 (ddd, *J* =9.6, 5.6, 2.0 Hz, 1H, H-5′), 3.85 (t, *J* =9.6 Hz, 1H, H-4′), 3.78 (dd, *J* =11.2, 5.6 Hz, 1H, H-6′), 3.71 (t, *J* = 8.8 Hz, 1H, H-3), 3.61–3.55 (m, 2H, H-2, H-4), 3.49 (ddd, *J* =9.6, 4.8, 1.6 Hz, 1H, H-5), 3.71 (s, 3H), 2.04 (s, 3H) ppm; ^{13}C NMR

(100 MHz, $CDCl_3$) δ 170.7 (C), 166.9 (C), 165.5 (C), 138.4 (C), 137.7 (C), 137.5 (C), 133.2 (CH), 133.0 (CH), 129.9 (CH), 129.7 (CH), 129.6 (CH), 129.2 (CH), 128.9 (CH), 128.5 (CH), 128.4 (CH), 128.3 (CH), 12.8.2 (CH), 128.1 (CH), 127.9 (CH), 127.8 (CH), 127.7 (CH), 125.3 (CH), 103.8 (CH), 96.9 (CH), 84.7 (CH), 82.1 (CH), 77.41 (CH), 76.6 (CH), 75.7 (CH_2), 75.0 (CH_2),74.6 (CH_2), 73.0 (CH), 72.6 (CH), 72.2 (CH), 69.7 (CH), 68.3 (CH_2), 63.0 (CH_2),55.3 (CH_3), 20.8 (CH_3) ppm; HRMS (ESI) *m*/*z* calcd for $C_{57}H_{58}O_{14}Na$ ($[M + Na]^+$) 989.3724, found 989.3734.

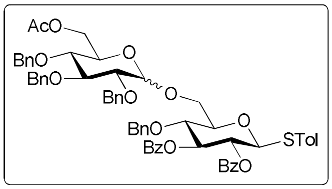

***p*-Tolyl 2,3-di-*O*-benzoyl-4-*O*-benzyl-6-(2,3,4-tri-*O*-benzyl-6-*O*-acetyl-D-glucopyranosyl)-1-thio-β-D-glucopyranoside (9i).**

According to the general procedure B for the glycosylation, glucopyranose **5** (98.5 mg, 0.200 mmol) in 1:1mixture of DCE and toluene (2.0 mL) was subjected to reaction with monosaccharide acceptor **2i** (233.9 mg, 0.4000 mmol) and DPAT **3b** (6.5 mg, 0.020 mmol) under microwave irradiation at 70 °C for 60 min. The crude product was purified by flash column chromatography to afford compound **9i** (122.9 mg, 58%, α:β = 3:1).

9i: Colorless oil; $[\alpha]^{28}_{D}$ +36.0 (*c* 2.83, CH_2Cl_2); IR (CH_2Cl_2) ν 1730, 1271, 1085, 1068, 1027, 710, 699 cm^{-1}; ^1H NMR (400 MHz, $CDCl_3$) δ 8.0 (d, *J* = 7.2 Hz, 2H, Ar*H*), 7.82 (d, *J* = 7.2 Hz, 2H, Ar*H*), 7.514–7.31 (m, 23H, Ar*H*), 7.16–7.08 (m, 7H, Ar*H*), 5.74 (t, *J* = 9.6 Hz, 1H, H-3′), 5.36 (t, *J* = 9.6 Hz, 1H, H-2′), 5.20 (d, *J* = 3.2 Hz, 1H, H-1), 5.11 (d, *J* = 10.8 Hz, 1H, ArC*H*), 4.96 (d, *J* = 11.2 Hz, 1H, ArC*H*), 4.91 (d, *J* = 10.8 Hz, 1H, ArC*H*), 4.87 (d, *J* = 9.6 Hz, 1H, H-1′), 4.72 (s, 2H, ArC*H*), 4.66 (d, *J* = 11.2 Hz, 1H, ArC*H*), 4.60 (d, *J* = 10.8 Hz, 1H, ArC*H*), 4.36 (m, 2H), 4.11 (t, *J* =

9.6 Hz, 1H, H-4′), 4.04–3.97 (m, 4H), 3.77 (ddd *J* =9.6, 7.2, 2.0 Hz, 1H, H-5′), 3.65 (dd *J* =10, 3.2 Hz, 1H, H-2), 3.61 (t, *J* = 10.0 Hz, 1H, H-4), 2.26 (s, 3H), 2.09 (s, 3H) ppm; ^{13}C NMR (100 MHz, $CDCl_3$) δ 170.6 (C), 165.5 (C), 165.1 (C), 138.5 (C), 138.4 (C), 137.9 (C), 137.2 (C), 134.0 (CH), 133.0 (CH), 129.7 (CH), 129.6 (CH), 129.3 (CH), 128.5 (CH), 128.3 (CH), 128.2 (CH), 128.1 (CH), 128.0 (CH), 127.9 (CH), 127.8 (CH), 127.7 (CH), 127.6 (CH), 96.8 (CH), 86.4 (CH), 81.7 (CH), 80.2 (CH), 79.6 (CH), 77.3 (CH), 76.2 (CH), 75.6 (CH_2), 75.0 (CH), 74.5 (CH_2), 72.7 (CH_2), 70.9 (CH), 68.8 (CH), 65.0 (CH_2), 63.0 (CH_2), 21.0 (CH_3), 20.8 (CH_3) ppm; HRMS (ESI) *m*/*z* calcd for $C_{63}H_{62}O_{13}NaS$ ($[M + Na]^+$) 1081.3809, found 1081.3810.

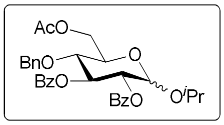

Isoproropyl 2,3-di-*O*-benzoyl-4-*O*-benzyl-6-*O*-acetyl-D-glucopyranoside (10d).

According to the general procedure B for the glycosylation, glucopyranose **6** (104.0 mg, 0.1998 mmol) in 1:1mixture of DCE and toluene (2.0 mL) was subjected to reaction with isopropanol (**2d**) (18 μL, 0.24 mmol) and DPAT **3b** (6.5 mg, 0.020 mmol) under microwave irradiation at 80 °C for 30 min. The crude product was purified by flash column chromatography to afford compound **10d** (79.9 mg, 71%, *α*:β = 1:2).

***α*-10d**: Colorless oil; $[\alpha]^{26}_{D}$ +98.8 (*c* 1.60, CH_2Cl_2); IR (CH_2Cl_2) ν 3064, 3032, 2973, 2921, 1724, 1261, 1095, 1069, 1027, 710 cm^{-1}; ^{1}H NMR (400 MHz, $CDCl_3$) δ 7.97 (m, 4H, Ar*H*), 7.48 (m, 2H, Ar*H*), 7.37 (m, 4H, Ar*H*), 7.18 (m, 6H, Ar*H*), 6.05 (dd, *J* =10.0, 9.6 Hz, 1H, H-3), 5.30 (d, *J* =3.6 Hz, 1H, H-1), 5.11 (dd, *J* =10.0, 3.6 Hz, 1H, H-2), 4.60 (d, *J* =10.8 Hz, 1H, ArC*H*), 4.52 (d, *J* =10.8 Hz, 1H, ArC*H*), 4.37 (dd, *J* =12.0, 2.4 Hz, 1H, H-6), 4.32 (dd, *J* =12.0, 4.0 Hz, 1H, H-6), 4.18 (ddd, *J* =9.6, 4.0, 2.4 Hz, 1H, H-5), 3.85 (quint, *J* =6.4 Hz, 1H, CH), 3.82 (t, *J* =9.6 Hz, 1H, H-4),

2.09 (s, 3H, CH_3), 1.22 (d, *J* =6.4 Hz, 3H, CH_3), 1.03 (d, *J* =6.4 Hz, 3H, CH_3) ppm; ^{13}C NMR (100 MHz, $CDCl_3$) δ 170.9 (C), 166.2 (C), 165.8 (C), 137.3 (C), 133.6 (CH), 133.4 (CH), 130.1 (CH), 130.0 (CH), 128.6 (CH), 128.6 (CH), 128.5 (CH), 128.4 (CH), 128.2 (CH), 94.9 (C-1), 76.3 (CH), 74.9 (CH_2), 73.3 (CH), 72.4 (CH), 71.8 (CH), 68.8 (CH), 63.1 (CH_2), 23.4 (CH_3), 22.0 (CH_3), 21.1 (CH_3) ppm; HRMS (ESI) *m/z* calcd for $C_{32}H_{34}O_9Na$ ([M + Na]$^+$) 585.2101, found 585.2095.

β-10d: Colorless oil; $[\alpha]^{26}_D$ +32.2 (*c* 2.25, CH_2Cl_2); IR (CH_2Cl_2) ν 3065, 3032, 2973, 1724, 1273, 1095, 1069, 1028, 710 cm^{-1}; ^{1}H NMR (400 MHz, $CDCl_3$) δ 7.93 (m, 4H, Ar*H*), 7.47 (m, 2H, Ar*H*), 7.35 (m, 4H, Ar*H*), 7.12 (m, 5H, Ar*H*), 5.71 (dd, *J* =10.0, 9.6 Hz, 1H, H-3), 5.30 (dd, *J* =10.0, 8.0 Hz, 1H, H-2), 4.73 (d, *J* =8.0 Hz, 1H, H-1), 4.57 (d, *J* =10.8 Hz, 1H, ArC*H)*, 4.49 (d, *J* =10.8 Hz, 1H, ArC*H)*, 4.42 (dd, *J* =12.0, 2.0 Hz, 1H, H-6), 4.26 (dd, *J* =12.0, 4.8 Hz, 1H, H-6), 3.91 (quint, *J* =6.4 Hz, 1H, CH), 3.85 (t, *J* =9.6 Hz, 1H, H-4), 3.73 (ddd, *J* =9.6, 4.8, 2.0 Hz, 1H, H-5), 2.08 (s, 3H, CH_3), 1.19 (d, *J* =6.4 Hz, 3H, CH_3), 1.02 (d, *J* =6.4 Hz, 3H, CH_3) ppm; ^{13}C NMR (100 MHz, $CDCl_3$) δ 170.9 (C), 165.9 (C), 165.4 (C), 137.1 (C), 133.4 (CH), 133.2 (CH), 129.9 (CH), 129.8 (CH), 128.6 (CH), 128.5 (CH), 128.4 (CH), 128.2 (CH), 100.1 (C-1), 76.1 (CH), 75.5 (CH), 74.9 (CH_2), 73.2 (CH), 72.4 (CH), 63.1 (CH_2), 23.4 (CH_3), 22.2 (CH_3), 21.1 (CH_3) ppm; HRMS (ESI) *m/z* calcd for $C_{32}H_{34}O_9Na$ ([M + Na]$^+$) 585.2101, found 585.2100.

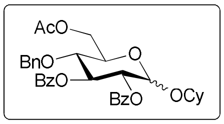

Cyclohexyl 2,3-di-*O*-benzoyl-4-*O*-benzyl-6-*O*-acetyl-D-glucopyranoside (10e).

According to the general procedure B for the glycosylation, glucopyranose **6** (104.0 mg, 0.1998 mmol) in 1:1mixture of DCE and toluene (2.0 mL) was subjected to reaction with cyclohexanol (**2e**) (25 μL, 0.24 mmol) and DPAT **3b**

(6.5 mg, 0.020 mmol) under microwave irradiation at 80 °C for 30 min. The crude product was purified by flash column chromatography to afford compound **10e** (95.0 mg, 79%, α:β = 1:2).

***α*-10e**: Colorless oil; $[\alpha]^{26}_{D}$ +99.0 (*c* 1.65, CH_2Cl_2); IR (CH_2Cl_2) ν 3062, 2934, 2858, 1724, 1263, 1094, 1068, 1025, 734, 708 cm^{-1}; ^{1}H NMR (400 MHz, $CDCl_3$) δ 7.97 (m, 4H, Ar*H*), 7.47 (m, 2H, Ar*H*), 7.36 (m, 4H, Ar*H*), 7.14 (m, 5H, Ar*H*), 6.06 (dd, *J* =10.0, 9.6 Hz, 1H, H-3), 5.34 (d, *J* =4.0 Hz, 1H, H-1), 5.12 (dd, *J* =10.0, 4.0 Hz, 1H, H-2), 4.60 (d, *J* =10.8 Hz, 1H, ArC*H)*, 4.52 (d, *J* =10.8 Hz, 1H, ArC*H)*, 4.38 (dd, *J* =12.0, 2.4 Hz, 1H, H-6), 4.32 (dd, *J* =12.0, 4.0 Hz, 1H, H-6), 4.20 (ddd, *J* =9.6, 4.0, 2.4 Hz, 1H, H-5), 3.82 (t, *J* =9.6 Hz, 1H, H-4), 3.57 (hept, *J* =3.6 Hz, 1H, CH), 2.08 (s, 3H, CH_3), 1.52 (m, 10H, CH_2) ppm; ^{13}C NMR (100 MHz, $CDCl_3$) δ 170.9 (C), 166.2 (C), 165.8 (C), 137.2 (C), 133.4 (CH), 133.3 (CH), 130.0 (CH), 129.9 (CH), 128.6 (CH), 128.5 (CH), 128.4 (CH), 128.2 (CH), 94.8 (C-1), 77.1 (CH), 76.3 (CH), 74.9 (CH_2), 73.1 (CH), 72.4 (CH), 68.8 (CH), 63.0 (CH_2), 33.4 (CH_2), 31.6 (CH_2), 25.7 (CH_2), 24.0 (CH_2), 23.7 (CH_2), 21.0 (CH) ppm; HRMS (ESI) *m*/*z* calcd for $C_{35}H_{38}O_9Na$ ($[M + Na]^+$) 625.2414, found 625.2414.

β-10e: Colorless oil; $[\alpha]^{26}_{D}$ +32.2 (*c* 1.50, CH_2Cl_2); IR (CH_2Cl_2) ν 3063, 3032, 2932, 2856, 1724, 1246, 1093, 1068, 1026, 709 cm^{-1}; ^{1}H NMR (400 MHz, $CDCl_3$) δ 7.93 (m, 4H, Ar*H*), 7.48 (m, 2H, Ar*H*), 7.35 (m, 4H, Ar*H*), 7.17 (m, 4H, Ar*H*), 7.11 (m, 4H, Ar*H*), 5.71 (t, *J* =9.6 Hz, 1H, H-3), 5.32 (dd, *J* =9.6, 8.0 Hz, 1H, H-2), 4.76 (d, *J* =8.0 Hz, 1H, H-1), 4.57 (d, *J* =10.8 Hz, 1H, ArC*H)*, 4.49 (d, *J* =10.8 Hz, 1H, ArC*H)*, 4.41 (dd, *J* =12.0, 2.4 Hz, 1H, H-6), 4.27 (dd, *J* =12.0, 4.8 Hz, 1H, H-6), 3.58 (t, *J* =9.6 Hz, 1H, H-4), 3.72 (ddd, *J* =9.6, 4.8, 2.4 Hz, 1H, H-5), 3.63 (hept, *J* =3.6 Hz, 1H, CH), 2.08 (s, 3H, CH_3), 1.39 (m, 10H, CH_2) ppm; ^{13}C NMR (100 MHz, $CDCl_3$) δ 170.9 (C), 165.9 (C), 165.4 (C), 137.1 (C), 133.4 (CH), 133.2 (CH), 130.0 (CH), 129.9 (CH), 128.6 (CH), 128.5 (CH), 128.4 (CH), 128.3 (CH), 99.8 (C-1), 78.2 (CH),

76.2 (CH), 75.6 (CH), 74.9 (CH_2), 73.2 (CH), 72.4 (CH), 63.1 (CH_2), 33.4 (CH_2), 31.8 (CH_2), 25.6 (CH_2), 23.9(CH_2), 23.7 (CH_2), 21.1 (CH_3) ppm; HRMS (ESI) *m*/*z* calcd for $C_{35}H_{38}O_9Na$ ([M + Na]$^+$) 625.2414, found 625.2414.

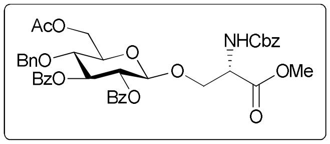

***O*-[2,3-D-*O*-Benzoyl-4-*O*-benzyl-6-*O*-acetyl-D-glucopyranosyl]-*N*-carbobenzyl-oxy-β-L-serine methyl ester (10f).**

According to the general procedure B for the glycosylation, glucopyranose **6** (104.0 mg, 0.1998 mmol) in 1:1mixture of DCE and toluene (2.0 mL) was subjected to reaction with *N*-Cbz-L-serine methyl ester (**2f**) (101.0 mg, 0.4293 mmol) and DPAT **3b** (6.5 mg, 0.020 mmol) under microwave irradiation at 80 °C for 30 min. The crude product was purified by flash column chromatography to afford compound **10f** (91.0 mg, 60%, β only).

β-10f: Colorless oil; $[\alpha]^{26}_D$ +39.9 (*c* 0.63, CH_2Cl_2); IR (CH_2Cl_2) ν 3425, 3063, 2954, 2887, 1726, 1272, 1093, 1069, 1027, 711 cm^{-1}; ^{1}H NMR (400 MHz, $CDCl_3$) δ 7.91 (m, 4H, Ar*H*), 7.48 (m, 2H, Ar*H*), 7.35 (m, 5H, Ar*H*), 7.17 (m, 3H, Ar*H*), 7.11 (m, 2H, Ar*H*), 5.69 (dd, *J* =9.6, 9.2 Hz, 1H, H-3), 5.46 (d, *J* =8.0 Hz, 1H, NH), 5.03 (d, *J* =12.4 Hz, 1H, ArC*H)*, 4.95 (d, *J* =12.4 Hz, 1H, ArC*H)*, 4.64 (d, *J* =10.8 Hz, 1H, ArC*H)*, 4.55 (d, *J* =10.8 Hz, 1H, ArC*H)*, 4.48 (d, *J* =10.8 Hz, 1H, ArC*H)*, 4.41 (m, 2H), 4.24 (m, 2H), 3.82 (m, 2H), 3.69 (ddd, *J* =9.6, 4.4, 2.4 Hz, 1H, H-5), 3.64 (s, 3H, CH_3), 2.05 (s, 3H, CH_3) ppm; ^{13}C NMR (100 MHz, $CDCl_3$) δ 170.9 (C), 170.1 (C), 165.8 (C), 165.5 (C), 137.0 (C), 133.5 (CH), 133.4 (CH), 130.0 (CH), 129.4 (C), 129.3 (C), 128.7 (CH), 128.6 (CH), 128.5 (CH), 128.4 (CH), 128.3 (CH), 101.3 (C-1), 75.7 (CH), 75.1 (CH), 74.9 (CH_2), 73.4 (CH), 72.0 (CH), 69.4 (CH_2), 67.2 (CH_2), 62.7 (CH_2), 54.4 (CH), 52.8 (CH_3), 20.1 (CH_3) ppm; HRMS (ESI) *m*/*z*) calcd for

$C_{41}H_{41}NO_{13}Na$ ([M + Na]$^+$) 778.2476, found 778.2470.

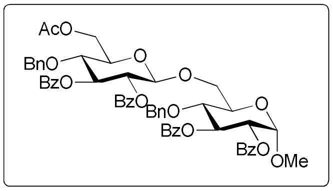

Methyl 2,3-di-*O*-benzoyl-4-*O*-benzyl-6-*O*-(2,3-di-O-benzoyl-4-*O*-benzyl-6-*O*-acetyl-D-glucopyranosyl)-β-D-glucopyranoside (10h).

According to the general procedure B for the glycosylation, glucopyranose **6** (104.0 mg, 0.1998 mmol) in 1:1mixture of DCE and toluene (2.0 mL) was subjected to reaction with monosaccharide acceptor **2h** (150.0 mg, 0.3046 mmol) and DPAT **3b** (6.5 mg, 0.020 mmol) under microwave irradiation at 70 °C for 60 min. The crude product was purified by flash column chromatography to afford compound **10h** (125.4 mg, 63%, β only).

β-10h: Colorless oil; $[\alpha]^{26}_{D}$ +96.3 (*c* 2.73, CH_2Cl_2); IR (CH_2Cl_2) ν 1721, 1262, 1093, 1068, 706 cm^{-1}; ^1H NMR (400 MHz, $CDCl_3$) δ 8.01–7.97 (m, 6H, Ar*H*), 7.98 (d, *J* = 7.2 Hz, 1H, Ar*H*), 7.54–7.50 (m, 3H, Ar*H*), 7.43–7.31 (m, 7H, Ar*H*), 7.29–7.17 (m, 10H, Ar*H*), 6.98–6.96 (m, 2H, Ar*H*), 5.98 (t, *J* = 9.6 Hz, 1H, H-3), 5.82 (t, *J* = 9.6 Hz, 1H, H-3′), 5.53 (dd, *J* =9.6, 8.0 Hz, 1H, H-2), 5.10–5.07(m, 2H, H-1′, H-2′), 4.75 (d, *J* = 8.0 Hz, 1H, H-1), 4.64 (d, *J* = 10.8 Hz, 1H, ArC*H*), 4.57 (d, *J* = 10.8 Hz, 1H, ArC*H*), 4.54 (dd, *J* =12.0, 2.0 Hz, 1H, H-6), 4.33 (dd, *J* =12.0, 3.6 Hz, 1H, H-6), 4.28 (s, 2H, ArC*H*), 4.24 (d, *J* =12.0, 1.6 Hz, 1H, H-6), 3.99–3.94 (m, 2H), 3.80 (m, 3H), 3.28 (s, 3H, CH_3), 2.07 (s, 3H, CH_3) ppm; ^{13}C NMR (100 MHz, $CDCl_3$) δ 170.6 (C), 165.8 (C), 165.6 (C), 165.4 (C), 165.1 (C), 137.1 (C), 136.8 (C), 133.3 (CH), 133.0 (CH), 132.9 (CH), 129.8 (CH), 129.7 (CH), 129.5 (CH), 129.3 (CH), 129.2 (CH), 128.4 (CH), 128.3 (CH), 128.2 (CH), 128.1 (CH), 127.6 (CH), 127.5 (CH), 101.2 (C-1), 96.8 (C-1′), 76.1 (CH), 75,7 (CH), 75.0 (CH), 74.7 (CH_2), 75.5 (CH_2),

73.2 (CH), 72.6 (CH), 72.2 (CH), 72.0 (CH), 69.4 (CH), 68.0 (CH_2), 62.6 (CH_2), 55.1 (CH_3), 20.9 (CH_3) ppm; HRMS (ESI) *m/z* calcd for $C_{57}H_{54}O_{16}Na$ ([M + Na]$^+$) 1017.3310, found 1017.3303.

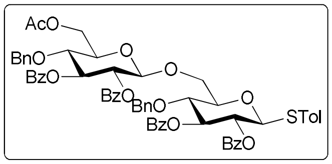

***p*-Tolyl 2,3-di-*O*-benzoyl-4-*O*-benzyl-6-(2,3-di-*O*-benzoyl-4-*O*-benzyl-6-*O*-acetyl-β-D-glucopyranosyl)-1-thio-β-D-glucopyranoside (10i).**

According to the general procedure B for the glycosylation, glucopyranose **6** (104.0 mg, 0.1998 mmol) in 1:1mixture of DCE and toluene (2.0 mL) was subjected to reaction with monosaccharide acceptor **2i** (233.9 mg, 0.4000 mmol) and DPAT **3b** (6.5 mg, 0.020 mmol) under microwave irradiation at 70 °C for 60 min. The crude product was purified by flash column chromatography to afford compound **10i** (121.8 mg, 56%, β only).

β-10i: Colorless oil; $[\alpha]^{26}_{D}$ +26.4 (*c* 0.95, CH_2Cl_2); IR (CH_2Cl_2) ν 3063, 3031, 2923, 2873, 1725, 1269, 1093, 1089, 1068, 1026, 737 cm^{-1}; ^{1}H NMR (400 MHz, $CDCl_3$) δ 7.99 (m, 2H, Ar*H*), 7.91 (m, 4H, Ar*H*), 7.83 (m, 2H, Ar*H*), 7.48 (m, 4H, Ar*H*), 7.35 (m, 10H, Ar*H*), 7.21 (m, 3H, Ar*H*), 7.17 (m, 4H, Ar*H*), 7.11 (m, 3H, Ar*H*), 6.95 (m, 2H, Ar*H*), 5.75 (dd, *J* =9.6, 9.2 Hz, 1H, H-3), 5.62 (dd, *J* =9.6, 9.2 Hz, 1H, H-3′), 5.47 (dd, *J* =9.6, 8.0 Hz, 1H, H-2), 5.28 (t, *J* =9.6 Hz, 1H, H-2′), 4.80 (d, *J* =8.0 Hz, 1H, H-1), 4.77 (d, *J* =9.6 Hz, 1H, H-1′), 4.59 (d, *J* =12.4 Hz, 1H, ArC*H)*, 4.56 (d, *J* =12.4 Hz, 1H, ArC*H)*, 4.51 (dd, *J* =12.0, 2.0 Hz, 1H, H-6), 4.32 (d, *J* =12.4 Hz, 1H, ArC*H*), 4.30 (dd, *J* =12.0, 4.0 Hz, 1H, H-6), 4.17 (dd, *J* =11.6, 1.2 Hz, 1H, H-6′), 3.95 (dd, *J* =9.6, 9.2 Hz, 1H, H-4), 3.86 (dd, *J* =11.6, 3.6 Hz, 1H, H-6′), 3.73 (m, 2H, H-5, H-4′), 3.65 (ddd, *J* =9.6, 3.6, 1.2 Hz, 1H, H-5′), 2.33 (s, 3H, CH_3), 2.08 (s, 3H, CH_3) ppm; ^{13}C NMR (100 MHz, $CDCl_3$) δ 170.9 (C), 165.8 (C), 165.4 (C), 138.7

(C), 137.3 (C), 137.1 (C), 133.8 (CH), 133.6 (CH), 133.5 (CH), 133.3 (CH), 130.1 (CH), 130.0 (CH), 129.9 (CH), 129.6 (CH), 128.6 (CH), 128.5 (CH), 128.4 (CH), 128.3 (CH), 127.9 (CH), 101.1 (C-1), 86.4 (C-1′), 79.1 (CH), 76.6 (CH), 76.0 (CH), 75.9 (CH), 75.5 (CH), 74.9 (CH_2), 74.8 (CH_2), 73.4 (CH), 72.3 (CH), 70.9 (CH), 68.0 (CH_2), 62.8 (CH_2), 21.4 (CH_3), 21.0 (CH_3) ppm; HRMS (ESI) *m/z* calcd for $C_{63}H_{58}O_{15}NaS$ ($[M + Na]^+$) 1109.3394, found 1109.3398.

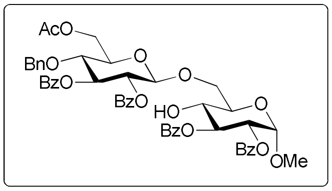

Methyl 2,3-di-*O*-benzoyl-6-*O*-(2,3-di-O-benzoyl-4-*O*-benzyl-6-*O*-acetyl-D-glucopyranosyl)-β-D-glucopyranosyl)-*α*-D-glucopyranoside (10j).

According to the general procedure B for the glycosylation, glucopyranose **6** (52.0 mg, 0.0999 mmol) in 1:1mixture of DCE and toluene (2.0 mL) was subjected to reaction with diol **2j** (72.0 mg, 0.179 mmol) and DPAT **3b** (6.5 mg, 0.020 mmol) under microwave irradiation at 70 °C for 60 min. The crude product was purified by flash column chromatography to afford compound **10j** (57.0 mg, 63%, β only).

β-10j: Colorless oil; $[\alpha]^{26}_D$ +71.6 (*c* 2.50, CH_2Cl_2); IR (CH_2Cl_2) ν 3489 (br), 1720, 1601, 1451, 1262, 1177, 1092, 1067, 1026, 993, 916, 853, 803, 736, 708 cm^{-1}; ^1H NMR (400 MHz, $CDCl_3$) δ 7.94 (m, 8H, Ar*H*), 7.57–7.30 (m, 10H, Ar*H*), 7.21–7.11 (m, 7H, Ar*H*), 5.76 (dd, *J* =9.6, 9.2 Hz, 1H, H-3), 5.61 (dd, *J* =10.0, 9.6 Hz, 1H, H-3′), 5.42 (dd, *J* = 9.6, 8.4 Hz, 1H, H-2), 5.08 (dd, *J* = 10.0, 3.6 Hz, 1H, H-2′), 4.96 (d, *J* = 3.6 Hz, 1H, H-1′), 4.82 (d, *J* = 8.4 Hz, 1H, H-1), 4.60 (d, *J* = 11.2 Hz, 1H, ArC*H*), 4.53 (d, *J* = 11.2 Hz, 1H, ArC*H*), 4.46 (d, *J* = 12.0 Hz, 1H, H-6), 4.27 (dd, *J* = 12.0, 4.0 Hz, 1H, H-6), 4.21 (d, *J* = 13.2 Hz, 1H, H-6′), 3.87 (m, 3H, H-4, H-5′, H-6′),

3.76 (m, 2H, H-4′, H-5), 3.22 (s, 3H), 2.95 (br, 1H, *O*H), 2.06 (s, 3H) ppm; ^{13}C NMR (100 MHz, $CDCl_3$) δ 170.7 (C), 167.2 (C), 165.8 (C), 165.3 (C), 136.8 (C), 133.3 (CH), 133.2 (CH), 133.1 (CH), 129.8 (CH), 129.7 (CH), 129.6 (CH), 129.3 (CH), 129.2 (CH), 128.4 (CH), 128.3 (CH), 128.2 (CH), 128.1 (CH), 128.0 (CH), 101.4 (C-1), 96.8 (C-1′), 75.7 (CH), 75.0 (CH), 74.7 (CH_2), 74.2 (CH), 73.2 (CH), 71.9 (CH), 71.3 (CH), 69.5 (CH), 68.5 (CH_2), 62.5 (CH_2), 55.1 (CH_3), 20.8 (CH_3) ppm; HRMS (ESI) *m*/*z* calcd for $C_{50}H_{48}O_{16}Na$ ($[M + Na]^+$) 927.2840, found 927.2862.

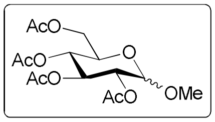

Methyl 2,3,4,6-*O*-tetraacetyl-glucopyranoside (11a)

According to the general procedure C for the glycosylation, glucopyranose **7** (70.2 mg, 0.202 mmol) in 1:1mixture of DCE and toluene (2.0 mL) was subjected to reaction with MeOH (**2a**) (25 μL, 0.62 mmol) and DPAT **3b** (6.2 mg, 0.019 mmol) under microwave irradiation at 80 °C for 30 min. Another portion of MeOH (25 μL, 0.62 mmol) was added and the reaction mixture was heated under microwave irradiation at 80 °C for 30 min. The reaction was workup and treated with pyridine and Ac_2O. The crude product was purified by flash column chromatography to afford compound **11a** as a white solid (42.1 mg, 58%, *α*:β = 1:1). The spectroscopic data of **11a** was in agreement with those previously reported in the literature.[20,21]

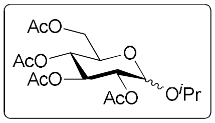

Isopropyl 2,3,4,6-*O*-tetraacetyl-D-glucopyranoside (11d).

According to the general procedure C for the glycosylation, glucopyranose **7** (68.7 mg, 0.197 mmol) in 1:1mixture of DCE and toluene (2.0 mL) was subjected

to reaction with isoproanol (**2d**) (20 μL, 0.26 mmol) and DPAT **3b** (6.9 mg, 0.022 mmol) under microwave irradiation at 80 °C for 30 min. Two other portions of isoproanol (20 μL x 2, 0.26 mmol x 2) were added and after each addition, the reaction mixture was heated under microwave irradiation at 80 °C for 30 min. The reaction was workup and treated with pyridine and Ac_2O. The crude product was purified by flash column chromatography to afford compound **11d** as a white solid (58.0 mg, 75%, α:β = 2:1). The spectroscopic data of **11d** was in agreement with those previously reported in the literature. [17,22]

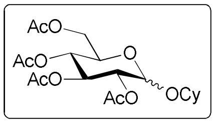

Cyclohexyl 2,3,4,6-*O*-tetraacetyl-D-glucopyranoside (11e).

According to the general procedure C for the glycosylation, glucopyranose **7** (69.2 mg, 0.199 mmol) in 1:1mixture of DCE and toluene (2.0 mL) was subjected to reaction with cyclohexanol (**2e**) (23.9 mg, 0.239 mmol) and DPAT **3b** (6.4 mg, 0.020 mmol) under microwave irradiation at 80 °C for 60 min. The reaction was workup and treated with pyridine and Ac_2O. The crude product was purified by flash column chromatography to afford compound **11e** as (53.8 mg, 62%, α:β = 2:1). The spectroscopic data of **11e** was in agreement with those previously reported in the literature. [22,23]

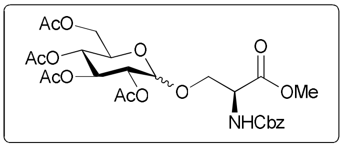

***O*-(2,3,4,6-*O*-Acetyl)-D-glucopyranosyl-*N*-benzyloxycarbonyl-L-serine-methyl ester (11f).**

According to the general procedure C for the glycosylation, glucopyranose **7** (69.8 mg, 0.200 mmol) in 1:1mixture of DCE and toluene (2.0 mL) was subjected to reaction with *N*-Cbz-L-serine methyl ester (**2f**) (60.5 mg, 0.239 mmol) and

DPAT **3b** (6.3 mg, 0.020 mmol) under microwave irradiation at 100 °C for 90 min. The reaction was workup and treated with pyridine and Ac_2O. The crude product was purified by flash column chromatography to afford compound **11f** as (45.3 mg, 39%, α:β = 1:1). The spectroscopic data of **β-11f** was in agreement with those previously reported in the literature.[24]

***α*-11f**: Colorless oil; $[\alpha]^{27}_{D}$ (*c* 0.23, $CHCl_3$); IR (CH_2Cl_2) *v* 3359 (NH), 2955, 2924, 1745 (C=O), 1523, 1368, 1221, 1036 cm^{-1}; ^1H NMR (500 MHz, $CDCl_3$) δ 7.38-7.29 (m, 5H, Ar-H), 5.73 (d, *J* = 8.4 Hz, 1H, NH), 5.37 (dd, *J* = 9.8, 9.8 Hz, 1H, H-3), 5.13 (s, 2H, CH_2Ph), 5.01-4.97 (m, 2H, H-1, H-4), 4.76 (dd, *J* = 10.0, 3.6 Hz, 1H, H-2), 4.53-4.51 (m, 1H), 4.19 (dd, *J* = 12.4, 4.7 Hz, 1H, H-6), 4.05 (dd, *J* = 12.4, 2.0 Hz, 1H, H-6), 3.99-3.91 (m, 3H, H-5), 3.74 (s, 3H, OMe), 2.05 (s, 3H, OAc), 2.02 (s, 3H, OAc), 2.00 (s, 3H, OAc), 1.98 (s, 3H, OAc) ppm; ^{13}C NMR (125 MHz, $CDCl_3$) δ 170.8 (C), 170.4 (C), 170.3 (x 2, C), 169.8 (C), 156.1 (C), 136.2 (C), 128.8 (CH), 128.5 (CH), 128.4(CH), 96.8 (CH, C-1), 70.9 (CH, C-2), 70.1 (CH, C-3), 69.4 (CH_2), 68.5 (CH, C-4), 67.9 (CH, C-5), 67.5 (CH_2), 61.9 (CH_2, C-6), 54.5 (CH), 52.9 (CH_3), 20.9 (x 2, CH_3), 20.8 (x 2, CH_3) ppm; HRMS (ESI) *m*/*z* calcd for $C_{26}H_{33}NO_{14}Na$ ($[M+Na]^+$) 606.1799, found 606.1802.

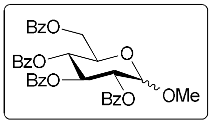

Methyl 2,3,4,6-*O*-tetrabenzoyl-D-glucopyranoside (12a).

According to the general procedure B for the glycosylation, glucopyranose **8** (118.7 mg, 0.1990 mmol) in 1:1mixture of DCE and toluene (2.0 mL) was subjected to reaction with MeOH (**2a**) (25 μL, 0.62 mmol) and DPAT **3b** (6.9 mg, 0.022 mmol) under microwave irradiation at 100 °C for 30 min. Three other portions of MeOH (25 μL x 3, 0.62 mmol x 3) were added and after each addition, the reaction mixture was heated under microwave irradiation at 100

°C for 30 min. The crude product was purified by flash column chromatography to afford compound **12a** as a white solid (77.5 mg, 64%, α:β = 1:2) and **2-OH-12a** (6.4 mg, 6.4%, α:β = 2:1). The spectroscopic data of **12a** was in agreement with those previously reported in the literature.[25,26]

Methyl 3,4,6-*O*-tribenzoyl-*α*-D-glucopyranoside (2-OH-*α*-12a): Colorless oil; $[\alpha]^{26}_{D}$ (*c* 0.23, $CHCl_3$); IR (CH_2Cl_2) *v* 3488 (OH), 2923, 1725 (C=O), 1602, 1451, 1270, 1096, 709 cm^{-1}; ^{1}H NMR (500 MHz, $CDCl_3$) δ 8.02-8.00 (m, 2H, Ar-H), 7.95-7.93 (m, 2H, Ar-H), 7.91-7.89 (m, 2H, Ar-H), 7.55-7.52 (m, 1H, Ar-H), 7.49-7.46 (m, 2H, Ar-H), 7.41-7.38 (m, 2H, Ar-H), 7.36-7.31 (m, 4H, Ar-H), 5.69 (dd, *J* = 9.8, 9.8 Hz, 1H, H-3), 5.55 (dd, *J* = 9.9, 9.9 Hz, 1H, H-4), 4.92 (d, *J* = 3.8 Hz, 1H, H-1), 4.56 (dd, *J* = 12.1, 2.8 Hz, 1H, H-6), 4.42 (dd, *J* = 12.1, 5.4 Hz, 1H, H-6), 4.30 (ddd, *J* = 5.2, 2.7 Hz, 1H, H-5), 3.90 (ddd, *J* = 10.9, 10.0, 3.8 Hz, 1H, H-2), 3.53 (s, 3H, OMe), 2.35 (d, *J* = 11.1 Hz, 1H, OH) ppm; ^{13}C NMR (125 MHz, $CDCl_3$) δ 167.0 (C), 166.4 (C), 165.5 (C), 133.7(C), 133.5 (C), 133.4 (C), 130.1 (CH), 130.0 (CH), 129.9 (CH), 129.8 (CH), 129.4 (CH), 129.0 (CH), 128.6 (x 2, CH), 128.5 (CH), 99.6 (CH, C-1), 74.1 (CH, C-3), 71.6 (CH, C-2), 69.1 (CH, C-4), 68.1 (CH, C-5), 63.2 (CH_2, C-6), 56.0 (CH_3) ppm; HRMS (ESI) *m*/*z* calcd for $C_{28}H_{26}O_9Na$ ([M+Na]$^+$) 529.1475, found 529.1472.

Methyl 3,4,6-*O*-tribenzoyl-*β*-D-glucopyranoside (2-OH-β-12a): Colorless oil; $[\alpha]^{26}_{D}$ 58.1 (*c* 0.07, $CHCl_3$); IR (CH_2Cl_2) *v* 3482 (OH), 2923, 2851, 1726 (C=O), 1602, 1451, 1270, 1115, 1069, 1027, 709 cm^{-1}; ^{1}H NMR (500 MHz, $CDCl_3$) δ 7.99-7.97 (m, 2H, Ar-H), 7.96-7.94 (m, 2H, Ar-H), 7.89-7.87 (m, 2H, Ar-H), 7.53-7.45 (m, 3H, Ar-H), 7.38-7.30 (m, 6H, Ar-H), 5.59-5.55 (m, 2H, H-3, H-4), 4.58 (dd, *J* = 12.0, 3.0 Hz, 1H, H-6), 4.48 (d, *J* = 7.7 Hz, 1H, H-1), 4.46 (dd, *J* = 11.9, 5.6 Hz, 1H, H-6), 4.05 (ddd, *J* = 10.0, 5.5, 3.5 Hz, 1H, H-5), 3.79 (m, 1H, H-2), 3.60 (s, 3H,

OMe), 2.66 (br s, 1H, OH) ppm; ^{13}C NMR (125 MHz, $CDCl_3$) δ 166.8 (C), 166.4 (C), 165.6 (C), 133.7 (C), 133.6 (C), 133.3 (C), 130.1 (CH), 130.0 (CH), 129.9 (CH), 129.9 (CH), 129.3 (CH), 129.1 (CH), 128.6 (CH), 128.6 (CH), 128.6 (CH), 104.2 (CH, C-1), 75.5 (CH, C-3), 73.1 (CH, C-2), 72.4 (CH, C-5), 69.7 (CH, C-4), 63.5 (CH_2, C-6), 57.7 (CH_3) ppm; HRMS (ESI) *m*/*z* calcd for $C_{28}H_{26}O_9Na$ ($[M+Na]^+$) 529.1475, found 529.1471.

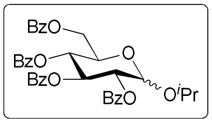

Isopropyl 2,3,4,6-*O*-tetrabenzoyl-D-glucopyranoside (12d).

According to the general procedure B for the glycosylation, glucopyranose **8** (119.1 mg, 0.1996 mmol) in 1:1mixture of DCE and toluene (2.0 mL) was subjected to reaction with isopropanol (**2d**) (20 μL, 0.26 mmol) and DPAT **3b** (6.6 mg, 0.021 mmol) under microwave irradiation at 100 °C for 30 min. Two other portions of isopropanol (20 μL x 2, 0.26 mmol x 2) were added and after each addition, the reaction mixture was heated under microwave irradiation at 100 °C for 30 min. The crude product was purified by flash column chromatography to afford compound **12d** (96.2 mg, 75%, *α*:β = 3:1) and **2-OH-12d** (5.2 mg, 5.1%, *α* only).

***α*-12d**: White solid; mp: 129-130 °C; $[\alpha]^{26}_D$ (*c* 0.21, $CHCl_3$); IR (CH_2Cl_2) *v* 3065, 2973, 1725 (C=O), 1602, 1452, 1269, 1094, 1041, 709 cm^{-1}; ^{1}H NMR (500 MHz, $CDCl_3$) δ 8.05-8.03 (m, 2H, Ar-H), 7.99-7.97 (m, 2H, Ar-H), 7.96-7.94 (m, 2H, Ar-H), 7.88-7.86 (m, 2H, Ar-H), 7.55-7.46 (m, 3H, Ar-H), 7.42-7.32 (m, 7H, Ar-H), 7.29-7.25 (m, 2H, Ar-H), 6.19 (dd, *J* = 9.9, 9.9 Hz, 1H, H-3), 5.67 (dd, *J* = 9.8, 9.8 Hz, 1H, H-4), 5.46 (d, *J* = 3.9 Hz, 1H, H-1), 5.27 (dd, *J* = 10.1, 3.9 Hz, 1H, H-2), 4.59 (dd, *J* = 11.7, 2.6 Hz, 1H, H-6), 4.55 (ddd, *J* = 10.0, 5.0, 2.6 Hz, 1H, H-5), 4.48 (dd, *J* = 11.7, 5.2 Hz, 1H, H-6), 3.93 (sept, *J* = 6.1 Hz, 1H, O*CH*Me_2),

1.29 (d, J = 6.1 Hz, 3H, OCHMe_2), 1208.2 (d, J = 6.1 Hz, 3H, OCHMe_2) ppm; ^{13}C NMR (125 MHz, $CDCl_3$) δ 166.4 (C), 166.0 (x 2, C), 165.5 (C), 133.5 (C), 133.5 (C), 133.2 (x 2, C), 130.0 (CH), 130.0 (CH), 129.9 (CH), 129.9 (CH), 129.4 (CH), 129.3 (CH), 129.1 (CH), 128.6 (CH), 128.6 (CH), 128.5 (CH), 128.4 (CH), 95.0 (CH, C-1), 72.3 (CH, C-2), 72.3 (CH), 70.8 (CH, C-3), 69.9 (CH, C-4), 68.0 (CH, C-5), 63.4 (CH_2, C-6), 23.4 (CH_3), 22.0 (CH_3) ppm; HRMS (ESI) *m/z* calcd for $C_{37}H_{34}O_{10}Na$ ([M+Na]$^+$) 661.2050, found 661.2058.

β-12d: White solid; mp: 110-111 °C; $[\alpha]^{26}_D$ (*c* 0.41, $CHCl_3$); IR (CH_2Cl_2) *v* 3064, 2973, 1721 (C=O), 1601, 1451, 1258, 1092, 1068, 1002, 707 cm^{-1}; ^{1}H NMR (500 MHz, $CDCl_3$) δ 8.00-7.98 (m, 2H, Ar-H), 7.95-7.93 (m, 2H, Ar-H), 7.89-7.87 (m, 2H, Ar-H), 7.83-7.81 (m, 2H, Ar-H), 7.54-7.46 (m, 3H, Ar-H), 7.43-7.31 (m, 7H, Ar-H), 7.28-7.25 (m, 2H, Ar-H), 5.88 (dd, J = 9.7, 9.7 Hz, 1H, H-3), 5.63 (dd, J = 9.8, 9.8 Hz, 1H, H-4), 5.48 (dd, J = 9.8, 7.9 Hz, 1H, H-2), 4.89 (d, J = 7.9 Hz, 1H, H-1), 4.61 (dd, J = 12.0, 3.2 Hz, 1H, H-6), 4.48 (dd, J = 12.0, 5.6 Hz, 1H, H-6), 4.14 (ddd, J = 10.0, 5.5, 3.2 Hz, 1H, H-5), 3.96 (sept, J = 6.2 Hz, 1H, O*CH*Me_2), 1.21 (d, J = 6.1 Hz, 3H, OCHMe_2), 1.05 (d, J = 6.2 Hz, 3H, OCHMe_2) ppm; ^{13}C NMR (125 MHz, $CDCl_3$) δ 166.4 (C), 166.1 (C), 165.4 (C), 165.2 (C), 133.6 (C), 133.4 (C), 133.4 (C), 133.3 (C), 130.0 (CH), 130.0 (CH), 129.9 (x 2, CH), 129.8 (CH), 129.6 (CH), 129.0 (CH), 128.9 (CH), 128.6 (CH), 128.6 (x 2, CH), 128.5 (CH), 100.3 (CH, C-1), 73.6, 73.1 (CH, C-3), 72.2 (CH, C-5), 70.1 (CH, C-2), 63.6 (CH, C-4), 63.6 (CH_2, C-6), 23.4 (CH_3), 22.2 (CH_3) ppm; HRMS (ESI) *m/z* calcd for $C_{37}H_{34}O_{10}Na$ ([M+Na]$^+$) 661.2050, found 661.2045.

Isopropyl 3,4,6-*O*-tribenzoyl-*α*-D-glucopyranoside (2-OH-*α*-12d): White solid; mp: 133-134 °C; $[\alpha]^{26}_D$ (*c* 0.23, $CHCl_3$); IR (CH_2Cl_2) *v* 3494 (OH), 3066, 2973, 2930, 1722 (C=O), 1602, 1452, 1269, 1095, 1026, 709 cm^{-1}; ^{1}H NMR (500 MHz, $CDCl_3$) δ 8.01-7.99 (m, 2H, Ar-H), 7.96-7.94 (m, 2H, Ar-H), 7.92-7.90

(m, 2H, Ar-H), 7.54-7.51 (m, 1H, Ar-H), 7.48-7.45 (m, 2H, Ar-H), 7.40-7.37 (m, 2H, Ar-H), 7.36-7.30 (m, 4H, Ar-H), 5.68 (dd, *J* = 9.8, 9.8 Hz, 1H, H-3), 5.52 (dd, *J* = 9.8, 9.8 Hz, 1H, H-4), 5.10 (d, *J* = 4.0 Hz, 1H, H-1), 4.56-4.52 (m, 1H, H-6), 4.44-4.37 (m, 2H, H-5, H-6), 4.00 (sept, *J* = 6.2 Hz, 1H, O*CH*Me2), 3.86 (ddd, *J* = 11.5, 9.9, 3.9 Hz, 1H, H-2), 2.20 (d, *J* = 11.6 Hz, 1H, OH), 1.32 (d, *J* = 6.2 Hz, 3H, OCH*Me2*), 1.23 (d, *J* = 6.2 Hz, 3H, OCH*Me2*) ppm; ^{13}C NMR (125 MHz, $CDCl_3$) δ 167.0 (C), 166.4 (C), 165.6 (C), 133.6 (C), 133.4 (C), 133.3 (C), 130.0 (CH), 129.9 (CH), 129.6 (CH), 129.2 (CH), 128.6 (CH), 128.6 (CH), 128.5 (CH), 97.4 (CH, C-1), 74.2 (CH, C-3), 72.1 (CH), 71.4 (CH, C-2), 69.4 (CH, C-4), 68.3 (CH, C-5), 63.5 (CH_2, C-6), 23.6 (CH_3), 22.2 (CH_3) ppm; HRMS (ESI) *m*/*z* calcd for $C_{30}H_{30}O_9Na$ ($[M+Na]^+$) 557.1788, found 557.1793.

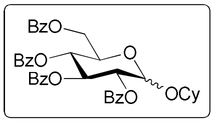

Cyclohexyl 2,3,4,6-*O*-tetrabenzoyl-D-glucopyranoside (12e).

According to the general procedure B for the glycosylation, glucopyranose **8** (119.0 mg, 0.1995 mmol) in 1:1mixture of DCE and toluene (2.0 mL) was subjected to reaction with cyclohexanol (**2e**) (25.0 mg, 0.250 mmol) and DPAT **3b** (6.8 mg, 0.021 mmol) under microwave irradiation at 100 °C for 60 min. The crude product was purified by flash column chromatography to afford compound **12e** as a white solid (96.0 mg, 71%, α:β = 6:1).

***α*-12e**: White solid; mp: 169-171 °C; $[\alpha]^{26}_D$ (*c* 0.27, $CHCl_3$); IR (CH_2Cl_2) *v* 3064, 2933, 2857, 1720 (C=O), 1601, 1451, 1261, 1091, 1025, 706 cm^{-1}; ^{1}H NMR (500 MHz, $CDCl_3$) δ 8.04-8.02 (m, 2H, Ar-H), 7.98-7.96 (m, 2H, Ar-H), 7.95-7.93 (m, 2H, Ar-H), 7.87-7.85 (m, 2H, Ar-H), 7.55-7.47 (m, 3H, Ar-H), 7.42-7.33 (m, 7H, Ar-H), 7.29-7.26 (m, 2H, Ar-H), 6.18 (dd, *J* = 9.6, 9.6 Hz, 1H, H-3), 5.63 (dd, *J* = 10.0, 10.0 Hz, 1H, H-4), 5.48 (d, *J* = 3.9 Hz, 1H, H-1), 5.25 (dd, *J* = 10.0, 3.6 Hz,

1H, H-2), 4.59-4.54 (m, 2H, H-5, H-6), 4.45 (dd, *J* = 12.5, 6.3 Hz, 1H, H-6), 3.63-3.57 (m, 1H), 1.96-1.92 (m, 1H), 1.71-1.58 (m, 3H), 1.54-1.41 (m, 2H), 1.29-1.08 (m, 4H) ppm; ^{13}C NMR (125 MHz, $CDCl_3$) δ 166.4 (C), 166.0 (x 2, C), 165.6 (C), 133.6 (C), 133.5 (C), 133.3 (x 2, C), 130.1 (CH), 130.0 (CH), 129.9 (x 2, CH)), 129.9 (CH), 129.5 (CH), 129.4 (CH), 129.1 (CH), 128.6 (x 2, CH), 128.6 (CH), 128.5 (CH), 94.9 (CH, C-1), 77.8 (CH), 72.3 (CH, C-2), 70.9 (CH, C-3), 70.0 (CH, C-4), 68.0 (CH, C-5), 63.5 (CH_2, C-6), 33.7 (CH_2), 31.8 (CH_2), 25.6 (CH_2), 24.2 (CH_2), 24.0 (CH_2) ppm; HRMS (ESI) *m/z* calcd for $C_{40}H_{38}O_{10}Na$ ($[M+Na]^+$) 701.2363, found 701.2359.

β-12e: White solid; mp: 155-156 °C; $[\alpha]^{26}_{D}$ (*c* 0.32, $CHCl_3$); IR (CH_2Cl_2) *v* 2934, 2857, 1722 (C=O), 1601, 1451, 1260, 1092, 1068, 1026, 708 cm^{-1}; ^{1}H NMR (500 MHz, $CDCl_3$) δ 8.00-7.98 (m, 2H, Ar-H), 7.94-7.92 (m, 2H, Ar-H), 7.89-7.87 (m, 2H, Ar-H), 7.83-7.81 (m, 2H, Ar-H), 7.53-7.45 (m, 3H, Ar-H), 7.42-7.31 (m, 7H, Ar-H), 7.28-7.25 (m, 2H, Ar-H), 5.88 (dd, *J* = 9.7, 9.7 Hz, 1H, H-3), 5.62 (dd, *J* = 9.6, 9.6 Hz, 1H, H-4), 5.49 (dd, *J* = 9.9, 7.9 Hz, 1H, H-2), 4.92 (d, *J* = 7.9 Hz, 1H, H-1), 4.60 (dd, *J* = 12.0, 3.4 Hz, 1H, H-6), 4.50 (dd, *J* = 12.0, 6.0 Hz, 1H, H-6), 4.13 (ddd, *J* = 9.9, 6.0, 3.2 Hz, 1H, H-5), 3.67-3.62 (m, 1H), 1.88-1.84 (m, 1H), 1.72-1.68 (m, 1H), 1.64-1.60 (m, 1H), 1.55-1.52 (m, 1H), 1.46-1.37 (m, 2H), 1.27-1.07 (m, 4H) ppm; ^{13}C NMR (125 MHz, $CDCl_3$) δ 166.3 (C), 166.1 (C), 165.5 (C), 165.3 (C), 133.6 (C), 133.4 (C), 133.3 (x 2, C), 130.0 (CH), 130.0 (CH), 129.9 (CH), 129.9 (x 2, CH), 129.7 (CH), 129.1 (CH), 129.1 (CH), 128.6 (CH), 128.5 (x 2, CH), 128.5 (CH), 100.1 (CH, C-1), 78.7 (CH), 73.3 (CH, C-3), 72.3 (CH, C-2), 72.3 (CH, C-5), 70.3 (CH, C-4), 63.7 (CH_2, C-6), 33.5 (CH_2), 31.8 (CH_2), 25.6 (CH_2), 23.9 (CH_2), 23.8 (CH_2) ppm; HRMS (ESI) *m/z* calcd for $C_{40}H_{38}O_{10}Na$ ($[M+Na]^+$) 701.2363, found 701.2369.

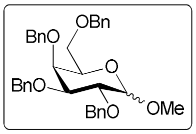

Methyl 2,3,4,6-tetra-*O*-benzyl-D-galactopyranoside (15a).

According to the general procedure B for the glycosylation, galactopyranose **13** (108.9 mg, 0.2014 mmol) in 1:1mixture of DCE and toluene (2.0 mL) was subjected to reaction with MeOH (**2a**) (25 μL, 0.62 mmol) and DPAT **3b** (6.3 mg, 0.020 mmol) under microwave irradiation at 60 °C for 30 min. The crude product was purified by flash column chromatography to afford compound **15a** (105.9 mg, 95%, α:β = 2:1). The spectroscopic data of **15a** was in agreement with those previously reported in the literature.[27]

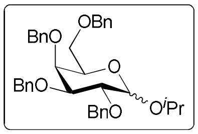

Isopropyl 2,3,4,6-tetra-*O*-benzyl-D-galactopyranoside (15d).

According to the general procedure B for the glycosylation, galactopyranose **13** (107.3 mg, 0.1985 mmol) in 1:1mixture of DCE and toluene (2.0 mL) was subjected to reaction with isopropanol (**2d**) (20 μL, 0.26 mmol) and DPAT **3b** (6.9 mg, 0.022 mmol) under microwave irradiation at 60 °C for 30 min. Another portion of isopropanol (20 μL, 0.26 mmol) was added and the reaction mixture was heated under microwave irradiation at 60 °C for 30 min. The crude product was purified by flash column chromatography to afford compound **15d** (94.3 mg, 82%, α:β = 2:1).

***α*-15d**: Colorless oil; $[\alpha]^{27}_{D}$ (*c* 0.27, $CHCl_3$); IR (CH_2Cl_2) *v* 3029, 2970, 2917, 2867, 1496, 1453, 1092, 1038, 733, 696 cm^{-1}; ^1H NMR (500 MHz, $CDCl_3$) δ 7.36-7.21 (m, 20H, Ar-H), 4.92 (d, *J* = 11.5 Hz, 1H, CH_2Ph), 4.90 (d, *J* = 4.0 Hz, 1H, H-1), 4.82 (d, *J* = 11.6 Hz, 1H, CH_2Ph), 4.77 (d, *J* = 11.9 Hz, 1H, CH_2Ph), 4.70 (d, *J*

= 11.6 Hz, 1H, CH_2Ph), 4.64 (d, *J* = 12.0 Hz, 1H, CH_2Ph), 4.54 (d, *J* = 11.5 Hz, 1H, CH_2Ph), 4.44 (d, *J* = 11.9 Hz, 1H, CH_2Ph), 4.37 (d, *J* = 11.9 Hz, 1H, CH_2Ph), 4.00-4.98 (m, 2H, H-2, H-6), 3.95 (d, *J* = 2.7 Hz, 1H, H-4), 3.92 (dd, *J* = 10.2, 2.9 Hz, 1H, H-3), 3.86 (sept, *J* = 6.2 Hz, 1H, O*CH*Me_2), 3.53-3.46 (m, 2H, H-5, H-6), 1.29 (d, *J* = 6.4 Hz, 3H, OCHMe_2), 1.15 (d, *J* = 6.0 Hz, 3H, OCHMe_2) ppm; ^{13}C NMR (125 MHz, $CDCl_3$) δ 139.2 (C), 139.0 (C), 138.9 (C), 138.3 (C), 128.6 (CH), 128.5 (CH), 128.5 (CH), 128.4 (CH), 128.4 (CH), 128.2 (CH), 128.1 (CH), 127.9 (CH), 127.9 (CH), 127.8 (CH), 127.7 (CH), 127.6 (CH), 95.7 (CH, C-1), 79.4 (CH, C-3), 76.7 (CH, C-2), 75.4 (CH, C-4), 74.9 (CH_2), 73.6 (CH_2), 73.4 (CH_2), 73.4 (CH_2), 69.3 (CH, C-5), 69.3 (CH), 69.3 (CH_2, C-6), 23.4 (CH_3), 21.5 (CH_3) ppm; HRMS (ESI) *m/z* calcd for $C_{37}H_{42}O_6Na$ ([M+Na]$^+$) 605.2879, found 605.2870.

β-15d: Colorless oil; $[\alpha]^{27}_D$ (*c* 0.33, $CHCl_3$); IR (CH_2Cl_2) *v* 3030, 2920, 2858, 1496, 1454, 1366, 1070, 734, 697 cm^{-1}; ^1H NMR (500 MHz, $CDCl_3$) δ 7.39-7.23 (m, 20H, Ar-H), 4.93-4.90 (m, 2H, CH_2Ph), 4.75-4.72 (m, 2H, CH_2Ph), 4.68 (d, *J* = 11.9 Hz, 1H, CH_2Ph), 4.60 (d, *J* = 11.8 Hz, 1H, CH_2Ph), 4.44-4.38 (m, 2H, CH_2Ph), 4.39 (d, *J* = 7.7 Hz, 1H, H-1), 3.97 (sept, *J* = 6.3 Hz, 1H, O*CH*Me_2), 3.85 (d, *J* = 2.7 Hz, 1H, H-4), 3.77 (dd, *J* = 9.6, 7.7 Hz, 1H, H-2), 3.56 (d, *J* = 6.2 Hz, 2H, H-6), 3.51-3.47 (m, 2H, H-3, H-5), 1.25 (d, *J* = 6.2 Hz, 3H, OCHMe_2), 1.20 (d, *J* = 6.1 Hz, 3H, OCHMe_2) ppm; ^{13}C NMR (125 MHz, $CDCl_3$) δ 139.1 (C), 138.9 (C), 138.8 (C), 138.2 (C), 128.6 (x 2, CH), 128.5 (CH), 128.5 (CH), 128.4 (CH), 128.3 (CH), 128.0 (x 2, CH), 128.0 (CH), 127.7 (x 3, CH), 102.7 (CH, C-1), 82.6 (CH, C-3), 79.8 (CH, C-2), 75.4 (CH_2), 74.6 (CH_2), 73.8 (CH, C-4), 73.7 (CH_2), 73.6 (CH, C-5), 73.3 (CH_2), 72.3 (CH_2), 69.3 (CH_2, C-6), 23.9 (CH_3), 22.4 (CH_3) ppm; HRMS (ESI) *m/z* calcd for $C_{37}H_{42}O_6Na$ ([M+Na]$^+$) 605.2879, found 605.2871.

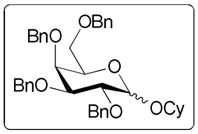

Cyclohexyl 2,3,4,6-tetra-*O*-benzyl-D-galactopyranoside (15e).

According to the general procedure B for the glycosylation, galactopyranose **13** (106.3 mg, 0.1966 mmol) in 1:1mixture of DCE and toluene (2.0 mL) was subjected to reaction with cyclohexanol (**2e**) (25.0 mg, 0.25 mmol) and DPAT **3b** (6.5 mg, 0.020 mmol) under microwave irradiation at 60 °C for 30 min. The crude product was purified by flash column chromatography to afford compound **15e** (77.3 mg, 59%, α:β = 2:1).

***α*-15e**: Colorless oil; $[\alpha]^{27}_{D}$ (*c* 0.34, $CHCl_3$); IR (CH_2Cl_2) *v* 3029, 2929, 2855, 1496, 1453, 1344, 1094, 1025, 732, 695 cm^{-1}; ^1H NMR (500 MHz, $CDCl_3$) δ 7.35-7.20 (m, 20H, Ar-H), 4.96 (d, *J* = 3.8 Hz, 1H, H-1), 4.91 (d, *J* = 11.4 Hz, 1H, *CH*$_2$Ph), 4.81 (d, *J* = 11.6 Hz, 1H, *CH*$_2$Ph), 4.74 (d, *J* = 11.9 Hz, 1H, *CH*$_2$Ph), 4.69 (d, *J* = 11.5 Hz, 1H, *CH*$_2$Ph), 4.63 (d, *J* = 12.0 Hz, 1H, *CH*$_2$Ph), 4.53 (d, *J* = 11.5 Hz, 1H, *CH*$_2$Ph), 4.44 (d, *J* = 11.8 Hz, 1H, *CH*$_2$Ph), 4.36 (d, *J* = 11.8 Hz, 1H, *CH*$_2$Ph), 4.01 (dd, *J* = 6.6, 6.6 Hz, 1H, H-6), 3.98 (dd, *J* = 9.8, 3.9 Hz, 1H, H-2), 3.94-3.90 (m, 2H, H-3, H-4), 3.52-3.47 (m, 3H, H-5, H-6), 1.88-1.81 (m, 2H), 1.74-1.66 (m, 2H), 1.51-1.47 (m, 1H), 1.44-1.36 (m, 1H), 1.35-1.27 (m, 1H), 1.24-1.09 (m, 3H) ppm; ^{13}C NMR (125 MHz, $CDCl_3$) δ 139.2 (C), 139.0 (x 2, C), 138.3 (C), 128.5 (CH), 128.5 (CH), 128.4 (CH), 128.4 (CH), 128.4 (CH), 128.1 (CH), 127.8 (x 2, CH), 127.7 (CH), 127.7 (CH), 127.6 (CH), 127.6 (CH), 95.6 (CH, C-1), 79.4 (CH, C-3), 76.8 (CH, C-2), 75.6 (CH, C-5), 75.4 (CH, C-4), 74.9 (CH_2), 73.6 (CH_2), 73.3 (CH_2), 73.2 (CH_2), 69.4 (CH), 69.3 (CH_2, C-6), 33.6 (CH_2), 31.8 (CH_2), 25.8 (CH_2), 24.7 (CH_2), 24.4 (CH_2) ppm; HRMS (ESI) *m*/*z* calcd for $C_{40}H_{46}O_6Na$ ($[M+Na]^+$) 645.3192, found 634.3194.

β-15e: Colorless oil; $[\alpha]^{27}{}_{D}$ (*c* 0.14, $CHCl_3$); IR (CH_2Cl_2) *v* 3054, 2935, 2859, 1496, 1453, 1362, 1028, 731, 700 cm^{-1}; ^1H NMR (500 MHz, $CDCl_3$) δ 7.35-7.21 (m, 20H, Ar-H), 4.94 (d, *J* = 10.7 Hz, 1H, CH_2Ph), 4.91 (d, *J* = 11.8 Hz, 1H, CH_2Ph), 4.74-4.71 (m, 2H, CH_2Ph), 4.68 (d, *J* = 11.6 Hz, 1H, CH_2Ph), 4.60 (d, *J* = 11.8 Hz, 1H, CH_2Ph), 4.43 (d, *J* = 7.7 Hz, 1H, H-1), 4.42 (d, *J* = 11.6 Hz, 1H, CH_2Ph), 4.39 (d, *J* = 11.6 Hz, 1H, CH_2Ph), 3.85 (d, *J* = 2.7 Hz, 1H, H-4), 3.78 (dd, *J* = 9.8, 7.8 Hz, 1H, H-2), 3.68-3.62 (m, 1H), 3.57-3.55 (m, 2H, H-6), 3.50-3.47 (m, 2H, H-3, H-5), 1.95-1.88 (m, 2H), 1.74-1.70 (m, 2H), 1.50-1.47 (m, 3H), 1.28-1.18 (m, 3H) ppm; ^{13}C NMR (125 MHz, $CDCl_3$) δ 139.1 (C), 138.9 (C), 138.8 (C), 138.2 (C), 128.6 (x 2, CH), 128.5 (CH), 128.5 (CH), 128.4 (CH), 128.4 (CH), 128.1 (CH), 128.0 (CH), 127.7 (x 2, CH), 127.7 (x 2, CH), 102.4 (CH, C-1), 82.7 (CH, C-3), 79.8 (CH, C-2), 77.7 (CH), 75.4 (CH_2), 74.7 (CH_2), 73.8 (CH, C-4), 73.7 (CH_2), 73.6 (CH, C-5), 73.3 (CH_2), 69.3 (CH_2, C-6), 33.9 (CH_2), 32.1 (CH_2), 25.9 (CH_2), 24.3 (CH_2), 24.2 (CH_2) ppm; HRMS (ESI) *m*/*z* calcd for $C_{40}H_{46}O_6Na$ ($[M+Na]^+$) 645.3192, found 634.3196.

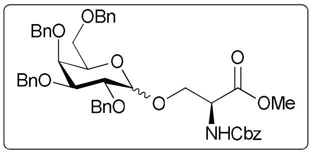

***O*-(2,3,4,6-*O*-Benzyl)-D-galactopyranosyl-*N*-benzyloxycarbonyl-L-serine-methyl ester (15f).**

According to the general procedure B for the glycosylation, galactopyranose **13** (108.1 mg, 0.1999 mmol) in 1:1mixture of DCE and toluene (2.0 mL) was subjected to reaction with *N*-Cbz-L-serine methyl ester (**2f**) (61.1 mg, 0.241 mmol) and DPAT **3b** (6.9 mg, 0.022 mmol) under microwave irradiation at 60 °C for 30 min. The crude product was purified by flash column chromatography to afford compound **15f** (86.8 mg, 56%, *α*:β = 2:1).

***α*-15f**: Colorless oil; $[\alpha]^{28}_D$ (*c* 0.24, $CHCl_3$); IR (CH_2Cl_2) *v* 3331 (NH), 3030, 2921, 1723 (C=O), 1497, 1497, 1454, 1345, 1209, 1096, 1055, 736, 697 cm^{-1}; ^1H NMR (500 MHz, $CDCl_3$) δ 7.30-7.17 (m, 25H, Ar-H), 6.12 (d, *J* = 8.9 Hz, 1H, NH), 5.03-4.98 (m, 2H, CH_2Ph), 4.84 (d, *J* = 11.3 Hz, 1H, CH_2Ph), 4.73-4.69 (m, 3H, H-1, CH_2Ph), 4.64 (d, *J* = 11.5 Hz, 1H, CH_2Ph), 4.54 (d, *J* = 11.9 Hz, 1H, CH_2Ph), 4.47 (d, *J* = 11.5 Hz, 1H, CH_2Ph), 4.45-4.43 (m, 1H), 4.39 (d, *J* = 12.0 Hz, 1H, CH_2Ph), 4.28 (d, *J* = 12.0 Hz, 1H, CH_2Ph), 4.11 (dd, *J* = 10.9, 3.5 Hz, 1H), 3.95 (dd, *J* = 10.0, 3.7 Hz, 1H, H-2), 3.87-3.85 (m, 2H, H-4, H-5), 3.78-3.73 (m, 2H, H-3), 3.60 (s, 3H, OMe), 3.45 (dd, *J* = 9.6, 6.4 Hz, 1H, H-6), 3.38 (dd, *J* = 9.5, 6.8 Hz, 1H, H-6) ppm; ^{13}C NMR (125 MHz, $CDCl_3$) δ 170.8 (C), 156.4 (C), 138.9 (C), 138.7 (C), 138.7 (C), 1381 (C), 136.5 (C), 128.7 (CH), 128.6 (x 2, CH), 128.5 (CH), 128.4 (CH), 128.3 (CH), 128.3 (CH), 128.0 (CH), 128.0 (CH), 127.9 (CH), 127.9 (CH), 127.9 (CH), 127.8 (CH), 127.7 (CH), 127.7 (CH), 99.7 (CH, C-1), 78.8 (CH, C-3), 76.6 (CH, C-2), 74.9 (CH, C-4), 74.9 (CH_2), 74.6 (CH_2), 73.5 (CH_2), 73.3 (CH_2), 70.7 (CH_2), 70.2 (CH, C-5), 69.1 (CH_2, C-6), 67.2 (CH_2), 54.9 (CH), 52.7 (CH_3) ppm; HRMS (ESI) *m/z* calcd for $C_{46}H_{49}NO_{10}Na$ ($[M+Na]^+$) 798.3254, found 798.3252.

***β*-15f**: White solid; mp: 78-80 °C; $[\alpha]^{27}_D$ (*c* 0.20, $CHCl_3$); IR (CH_2Cl_2) *v* 3355 (NH), 3030, 2921, 2853, 1724 (C=O), 1497, 1454, 1345, 1209, 1095, 1065, 736, 697 cm^{-1}; ^1H NMR (500 MHz, $CDCl_3$) δ 7.34-7.18 (m, 25H, Ar-H), 5.75 (d, *J* = 8.1 Hz, 1H, NH), 5.07-5.02 (m, 2H, CH_2Ph), 4.89 (d, *J* = 11.6 Hz, 1H, CH_2Ph), 4.79 (d, *J* = 11.0 Hz, 1H, CH_2Ph), 4.75-7.69 (m, 2H, CH_2Ph), 4.67 (d, *J* = 11.9 Hz, 1H, CH_2Ph), 4.57 (d, *J* = 11.6 Hz, 1H, CH_2Ph), 4.47-4.44 (m, 1H), 4.39 (d, *J* = 11.6 Hz, 1H, CH_2Ph), 4.35 (d, *J* = 11.7 Hz, 1H, CH_2Ph), 4.30-4.28 (m, 1H), 4.28 (d, *J* = 7.8 Hz, 1H, H-1), 3.85 (d, *J* = 2.2 Hz, 1H, H-4), 3.78-3.73 (m, 2H, H-2), 3.70 (s, 3H, OMe), 3.52-3.51 (m, 2H, H-6), 3.48-3.45 (m, 2H, H-3, H-5) ppm; ^{13}C NMR (125

MHz, $CDCl_3$) δ 170.6 (C), 156.3 (C), 138.7 (C), 138.5 (x 2, C), 138.0 (C), 136.5 (C), 128.7 (x 2, CH), 128.6 (CH), 128.6 (x 2, CH), 128.4 (CH), 128.4 (CH), 128.3 (CH), 128.1 (CH), 128.1 (CH), 127.9 (x 2, CH), 127.8 (CH), 127.8 (x 2, CH), 104.7 (CH, C-1), 82.4 (CH, C-3), 79.2 (CH, C-2), 75.6 (CH_2), 74.8 (CH_2), 73.7 (CH, C-5), 73.7 (CH_2), 73.5 (CH, C-4), 73.2 (CH_2), 70.2 (CH_2), 68.7 (CH_2, C-6), 67.2 (CH_2), 54.7 (CH), 52.9 (CH_3) ppm; HRMS (ESI) *m*/*z* calcd for $C_{46}H_{49}NO_{10}Na$ ($[M+Na]^+$) 798.3254, found 798.3253.

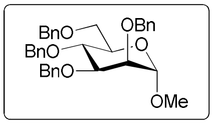

Methyl 2,3,4,6-tetra-*O*-benzyl-*α*-D-mannopyranoside (16a).

According to the general procedure B for the glycosylation, mannopyranose **14** (108.1 mg, 0.1999 mmol) in 1:1mixture of DCE and toluene (2.0 mL) was subjected to reaction with MeOH (**2a**) (25 μL, 0.62 mmol) and DPAT **3b** (6.6 mg, 0.021 mmol) under microwave irradiation at 60 °C for 30 min. The crude product was purified by flash column chromatography to afford compound **16a** (99.3 mg, 90%, *α* only). The spectroscopic data of **16a** was in agreement with those previously reported in the literature.[28]

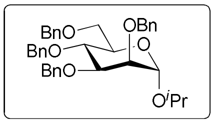

Isopropyl 2,3,4,6-tetra-*O*-benzyl-*α*-D-mannopyranoside (16d).

According to the general procedure B for the glycosylation, mannopyranose **14** (110.2 mg, 0.2038 mmol) in 1:1mixture of DCE and toluene (2.0 mL) was subjected to reaction with isopropanol (**2d**) (20 μL, 0.26 mmol) and DPAT **3b** (6.4 mg, 0.020 mmol) under microwave irradiation at 60 °C for 30 min. Another portion of isopropanol (20 μL, 0.26 mmol) was added and the reaction mixture was heated under microwave irradiation at 60 °C for 30 min. The crude product

was purified by flash column chromatography to afford compound **16d** (96.9 mg, 82%, α only). The spectroscopic data of **16d** was in agreement with those previously reported in the literature. [29]

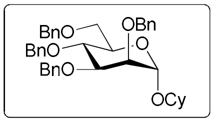

Cyclohexyl 2,3,4,6-tetra-*O*-benzyl-*α*-D-mannopyranoside (16e).

According to the general procedure B for the glycosylation, mannopyranose **14** (109.3 mg, 0.2022 mmol) in 1:1mixture of DCE and toluene (2.0 mL) was subjected to reaction with cyclohexanol (**2e**) (24.0 mg, 0.24 mmol) and DPAT **3b** (6.2 mg, 0.019 mmol) under microwave irradiation at 60 °C for 30 min. The crude product was purified by flash column chromatography to afford compound **16e** (91.6 mg, 73%, α only).

***α*-16e**: Colorless oil; $[\alpha]^{28}_{D}$ (*c* 0.38, $CHCl_3$); IR (CH_2Cl_2) v 3029, 2929, 2855, 1496, 1453, 1360, 1094, 1047, 1025, 733, 696 cm^{-1}; ^1H NMR (500 MHz, $CDCl_3$) δ 7.37-7.23 (m, 18H, Ar-H), 7.17-7.15 (m, 2H, Ar-H), 4.99 (d, J = 1.3 Hz, 1H, H-1), 4.87 (d, J = 10.7 Hz, 1H, CH_2Ph), 4.76 (d, J = 12.5 Hz, 1H, CH_2Ph), 4.69 (d, J = 12.4 Hz, 1H, CH_2Ph), 4.67-4.60 (m, 3H, CH_2Ph), 4.53 (d, J = 12.4 Hz, 1H, CH_2Ph), 4.50 (d, J = 10.8 Hz, 1H, CH_2Ph), 3.98 (dd, J = 9.3, 9.3 Hz, 1H, H-4), 3.93 (dd, J = 9.3, 2.7 Hz, 1H, H-3), 3.85 (ddd, J = 9.5, 4.6, 1.3 Hz, 1H, H-5), 3.79 (dd, J = 10.7, 5.1 Hz, 1H, H-6), 3.73-3.71 (m, 2H, H-2, H-6), 3.59-3.54 (m, 1H), 1.83-1.80 (m, 1H), 1.75-1.72 (m, 1H), 1.67-1.62 (m, 2H), 1.49-1.46 (m, 1H), 1.35-1.13 (m, 5H) ppm; ^{13}C NMR (125 MHz, $CDCl_3$) δ 138.8 (C), 138.7 (x 3, C), 128.5 (x 3, CH), 128.4 (CH), 128.3 (CH), 128.1 (CH), 127.9 (CH), 127.8 (CH), 127.8 (CH), 127.7 (CH), 127.7 (CH), 127.6 (CH), 95.9 (CH, C-1, $^1J_{CH}$ = 167.9 Hz), 80.5 (CH, C-3), 75.5 (CH, C-2), 75.4 (CH_2), 75.4 (CH, C-4), 74.9(CH), 73.5 (CH_2), 72.8 (CH_2), 72.4 (CH_2), 71.9 (CH, C-5), 69.6 (CH_2, C-6), 33.4 (CH_2), 31.5 (CH_2), 25.8 (CH_2), 24.2(CH_2), 24.0 (CH_2)

ppm; HRMS (ESI) *m*/*z* calcd for $C_{40}H_{46}O_6Na$ ([M+Na]$^+$) 645.3192, found 645.3186.

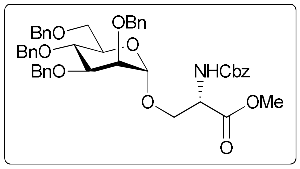

O-(2,3,4,6-O-Benzyl)-α-D-mannopyranosyl-N-benzyloxycarbonyl-L-serine-methyl ester (16f).

According to the general procedure B for the glycosylation, mannopyranose **14** (110.1 mg, 0.2036 mmol) in 1:1mixture of DCE and toluene (2.0 mL) was subjected to reaction with *N*-Cbz-L-serine methyl ester (**2f**) (64.0 mg, 0.253 mmol) and DPAT **3b** (6.6 mg, 0.021 mmol) under microwave irradiation at 60 °C for 30 min. The crude product was purified by flash column chromatography to afford compound **16f** (98.1 mg, 62%, α only).

α-16f: Colorless oil; $[\alpha]^{27}_D$ (*c* 0.29, $CHCl_3$); IR (CH_2Cl_2) v 3331 (NH), 3030, 2913, 1723 (C=O), 1497, 1454, 1209, 1056, 737, 697 cm^{-1}; ^{1}H NMR (500 MHz, $CDCl_3$) δ 7.34-7.24 (m, 23H, Ar-H), 7.14-7.12 (m, 2H, Ar-H), 5.73 (d, *J* = 8.5 Hz, 1H, NH), 5.10 (d, *J* = 12.2 Hz, 1H, CH_2Ph), 5.06 (d, *J* = 12.3 Hz, 1H, CH_2Ph), 4.81 (d, *J* = 10.8 Hz, 1H, CH_2Ph), 4.78 (d, *J* = 1.8 Hz, 1H, H-1), 4.71 (d, *J* = 12.4 Hz, 1H, CH_2Ph), 4.65 (d, *J* = 12.4 Hz, 1H, CH_2Ph), 4.63-4.57 (m, 3H, CH_2Ph), 4.50-4.45 (m, 3H, CH_2Ph), 3.96-3.91 (m, 2H, H-4, H-6), 3.86 (dd, *J* = 10.7, 3.4 Hz, 1H, H-6), 3.76 (dd, *J* = 9.0, 2.9 Hz, 1H, H-3), 3.71-3.67 (m, 2H, H-5), 3.67-3.64 (m, 5H, H-2, OMe) ppm; ^{13}C NMR (125 MHz, $CDCl_3$) δ 170.7 (C), 156.2 (C), 138.5 (C), 138.5 (C), 138.5 (C), 138.4 (C), 136.3 (C), 128.7 (CH), 128.6 (CH), 128.5 (CH), 128.5 (CH), 128.5 (x 2, CH), 128.4 (x 2, CH), 128.2 (x 2, CH), 128.0 (x 3, CH), 127.8 (CH), 127.7 (CH), 99.5 (CH, C-1, $^1J_{CH}$ = 169.2 Hz), 79.8 (CH, C-3), 75.2 (CH_2), 74.9 (CH, C-2), 74.9 (CH, C-4), 73.5(CH_2), 72.9(CH_2), 72.6 (CH, C-5), 72.6 (CH_2), 69.2 (CH_2), 69.1 (CH_2, C-6), 67.3 (CH_2), 54.5 (CH), 52.7 (CH_3) ppm; HRMS (ESI) *m*/*z* calcd for

$C_{46}H_{49}NO_{10}Na$ ([M+Na]$^+$) 798.3254, found 798.3251.

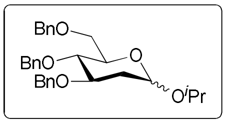

Isoproropyl 3,4,6-tri-*O*-benzyl-2-deoxy-D-glucopyranoside (19d).

According to the general procedure D for the glycosylation, 2-dexoyglucopyranose **17** (87.0 mg, 0.200 mmol) in DCE (2.0 mL) was subjected to reaction with isopropanol (**2d**) (18.0 mg, 0.300 mmol) and DPAT **3b** (6.5 mg, 0.020 mmol) at room temperature for 60 min. The crude product was purified by flash column chromatography to afford compound **19d** as a colorless oil (64.8 mg, 68%, α:β = 3:1). The spectroscopic data of **19d** was in agreement with those previously reported in the literature.[30,31]

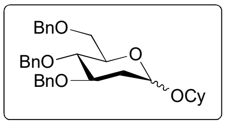

Cyclohexyl 3,4,6-tri-*O*-benzyl-2-deoxy-D-glucopyranoside (19e).

According to the general procedure D for the glycosylation, 2-dexoyglucopyranose **17** (87.0 mg, 0.200 mmol) in DCE (2.0 mL) was subjected to reaction with cyclohexanol (**2e**) (25.0 mg, 0.250 mmol) and DPAT **3b** (6.5 mg, 0.020 mmol) at room temperature for 60 min. The crude product was purified by flash column chromatography to afford compound **19e** as a colorless oil (61.6 mg, 60%, α:β = 3:1). The spectroscopic data of **19e** was in agreement with those previously reported in the literature.[31]

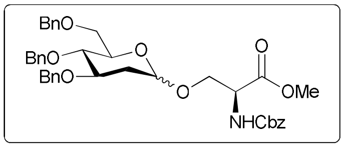

***O*-[3,4,6-Tri-*O*-benzyl-2-deoxy-D-glucopyranosyl]-*N*-carbobenzyloxy-L-serine methyl ester (19f)**.

According to the general procedure D for the glycosylation, 2-dexoyglucopyranose **17** (87.0 mg, 0.200 mmol) in DCE (2.0 mL) was subjected to reaction with *N*-Cbz-L-serine methyl ester (**2f**) (61.0 mg, 0.241 mmol) and DPAT **3b** (6.5 mg, 0.020 mmol) at room temperature for 60 min. The crude product was purified by flash column chromatography to afford compound **19f** as a colorless oil (68.1 mg, 52%, α:β = 5:1). The spectroscopic data of **19f** was in agreement with those previously reported in the literature.[32]

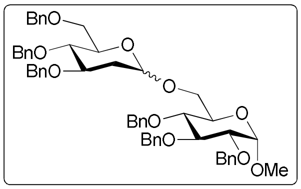

Methyl 2,3,4-tri-*O*-benzyl-6-*O*-(3,4,6-tri-*O*-benzyl-2-deoxy-D-glucopyranosyl)-α-D-glucopyranoside (19k).

According to the general procedure D for the glycosylation, 2-dexoyglucopyranose **17** (87.0 mg, 0.200 mmol) in DCE (2.0 mL) was subjected to reaction with monosaccharide acceptor **2k** (185.0 mg, 0.3982 mmol) and DPAT **3b** (6.5 mg, 0.020 mmol) at room temperature for 60 min. The crude product was purified by flash column chromatography to afford compound **19k** as a colorless oil (89.9 mg, 51%, α:β = 4:1). The spectroscopic data of **19k** was in agreement with those previously reported in the literature.[33]

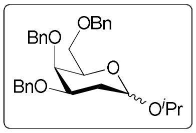

Isopropyl 3,4,6-tri-*O*-benzyl-2-deoxy-D-galactcopyranoside (20d).

According to the general procedure D for the glycosylation, 2-dexoygalcopyranose **18** (87.0 mg, 0.200 mmol) in DCE (2.0 mL) was subjected to reaction with isopropanol (**2d**) (18.0 mg, 0.300 mmol) and DPAT **3b** (6.5 mg,

0.020 mmol) at room temperature for 60 min. The crude product was purified by flash column chromatography to afford compound **20d** (66.7 mg, 70%, α:β = 6:1). The spectroscopic data of **20d** was in agreement with those previously reported in the literature.[30]

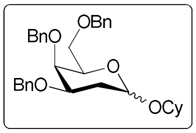

Cyclohexyl 3,4,6-tri-*O*-benzyl-2-deoxy-D-galactcopyranoside (20e).

According to the general procedure D for the glycosylation, 2-dexoygalcopyranose **18** (87.0 mg, 0.200 mmol) in DCE (2.0 mL) was subjected to reaction with cyclohexanol (**2e**) (25.0 mg, 0.250 mmol) and DPAT **3b** (6.5 mg, 0.020 mmol) at room temperature for 60 min. The crude product was purified by flash column chromatography to afford compound **20e** (75.0 mg, 73%, α:β = 6:1). The spectroscopic data of **20e** was in agreement with those previously reported in the literature.[34]

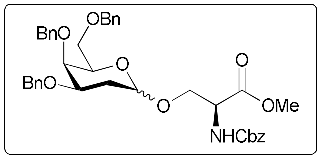

***O*-[3,4,6-Tri-*O*-benzyl-2-deoxy-D-galactopyranosyl]-*N*-carbobenzyloxy-L-serine methyl ester (20f).**

According to the general procedure D for the glycosylation, 2-dexoygalcopyranose **18** (87.0 mg, 0.200 mmol) in DCE (2.0 mL) was subjected to reaction with *N*-Cbz-L-serine methyl ester (**2f**) (61.0 mg, 0.241 mmol) and DPAT **3b** (6.5 mg, 0.020 mmol) at room temperature for 60 min. The crude product was purified by flash column chromatography to afford compound **20f** (89.1 mg, 68%, α:β = 10:1).

20f: Colorless oil; $[\alpha]^{26}_{D}$ +50.7 (*c* 2.07, CH_2Cl_2); IR (CH_2Cl_2) ν 1721, 1207, 1054,

1025, 734, 695 cm^{-1}; ^{1}H NMR (400 MHz, $CDCl_3$) δ 7.43–7.27 (m, 20 H, Ar*H*), 5.85 (d, *J* = 8.8 Hz, NH), 5.13 (m, 2H, ArC*H*), 4.94 (d, *J* = 3.6 Hz, 1H, H-1), 4.91 (d, *J* = 12.0 Hz, 1H, ArC*H*), 4.61–4.55 (m, 3 H, ArC*H*), 4.53–4.38 (m, 3H, CH, ArC*H*), 3.97 (dd, *J* =10.8, 3.6 Hz, 1H, CH*H'*CH), 3.89–3.82 (m, 4H, H-3, H-4, H-5, C*H*H'CH), 3.73 (s, 3H, CH_3), 3.59–3.52 (m, *J* = 10.8 Hz, 2H, H-6), 2.24 (dt, *J* = 12.4, 3.6 Hz, 1H, H-2ax), 1.91 (dd, *J* = 12.4, 4.4 Hz, 1H, H-2eq.) ppm; ^{13}C NMR (100 MHz, $CDCl_3$) δ 170.7 (C), 155.7 (C), 138.7 (C), 138.3 (C), 138.0 (C), 136.2 (C), 128.4 (CH), 128.3 (CH), 128.1 (CH), 127.7 (CH), 127.6 (CH), 127.5 (CH), 127.3 (CH), 99.0 (C-1), 74.2 (CH, CH_2), 73.3 (CH_2), 72.8 (CH), 70.4 (CH, CH_2), 69.4 (CH_2), 68.5 (CH_2), 67.0 (CH_2), 52.4 (CH_3), 30.9 (CH_2) ppm; HRMS (ESI) *m/z* calcd for $C_{39}H_{43}NO_9Na$ ([M + Na]$^+$) 692.2836, found 692.2831.

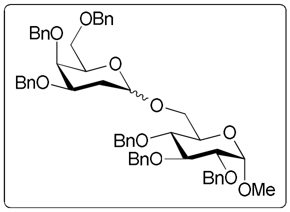

Methyl 2,3,4-tri-*O*-benzyl-6-*O*-(3,4,6-tri-*O*-benzyl-2-deoxy-D-galactopyranosyl)-*α*-D-glucopyranoside (20k).

According to the general procedure D for the glycosylation, 2-dexoygalcopyranose **18** (87.0 mg, 0.200 mmol) in DCE (2.0 mL) was subjected to reaction with monosaccharide acceptor **2k** (185.0 mg, 0.3982 mmol) and DPAT **3b** (6.5 mg, 0.020 mmol) at room temperature for 60 min. The crude product was purified by flash column chromatography to afford compound **20k** as a colorless oil (89.9 mg, 51%, *α*:β = 6:1). The spectroscopic data of **20k** was in agreement with those previously reported in the literature.[35]

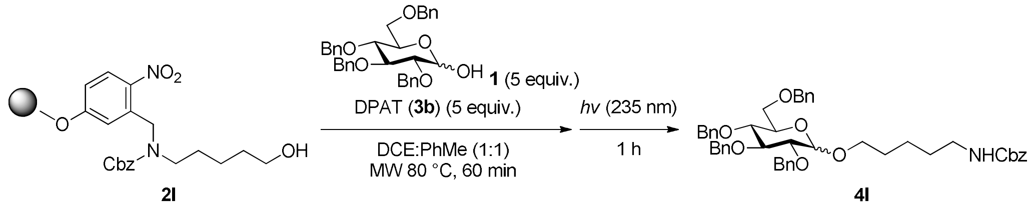

Merrifield resin modified with the photolabile *o*-nitrobenzyl alcohol linker **2l** (200 mg, 0.5 mmol/g) was swollen in CH_2Cl_2 for 1 hour. After draining CH_2Cl_2, the resin was mixed with glucopyranose **1** (270.0 mg, 0.4994 mmol) and DPAT **3b** (16.0 mg, 0.501 mmol) (16.0 mg, 0.501 mmol) in a 1:1 mixture of DCE and toluene (4.0 mL). The microwave-assisted reaction was carried out twice at 80 °C for 30 min. The crude products were obtained after photo cleavage (UV-235 nm) for 1 h and purified by flash column chromatography to afford **4l** (126.0 mg, 55%, α:β = 1:1).

α-4l: Colorless oil; $[\alpha]^{26}_{D}$ +59.3 (*c* 0.15, CH_2Cl_2); IR (CH_2Cl_2) ν 2919, 1717, 1586, 1453, 1240, 1026, 734, 696 cm^{-1}; ^{1}H NMR (500 MHz, $CDCl_3$) δ 7.31-7.24 (m, 23H, Ph-H), 7.12-7.10 (m, 2H, Ph-H), 5.06 (s, 2H, CH_2Ph), 4.96 (d, *J* = 10.90 Hz, 1H, CH_2Ph), 4.81 (d, *J* = 10.40 Hz, 1H, CH_2Ph), 4.79 (d, *J* = 10.70 Hz, 1H, CH_2Ph), 4.76 (d, *J* = 12.10 Hz, 1H, CH_2Ph), 4.71 (d, *J* = 3.45 Hz, 1H, H-1), 4.61 (d, *J* = 12.10 Hz, 1H, CH_2Ph), 4.57 (d, *J* = 12.10 Hz, 1H, CH_2Ph), 4.45 (d, *J* = 11.00 Hz, 2H, CH_2Ph), 3.95 (t, *J* = 9.25 Hz, 1H, H-3), 3.74 (d, *J* = 10.35 Hz, 1H, H-5), 3.69 (dd, *J* = 3.73, 10.48 Hz, 1H, H-6a), 3.63-3.60 (m, 2H, H-6b, GluOC*H*H), 3.60 (t, *J* = 9.65 Hz, 1H, H-4), 3.53 (dd, *J* = 3.58, 9.63 Hz, H-2), 3.37 (dt, *J* = 6.40, 9.60 Hz, 1H, GluOCH*H*), 3.15 (q, *J* = 6.53 Hz, 2H, CH_2), 1.66-1.50 (m, 2H, CH_2), 1.49 (quintet, 2H, CH_2), 1.37 (m, 2H, CH_2) ppm; ^{13}C NMR (125 MHz, $CDCl_3$) δ 156.36 (C), 138.84 (C), 138.27 (C), 138.18 (C), 137.90 (C), 136.64 (C), 128.46 (CH), 128.40 (CH), 128.34 (CH), 128.08 (CH), 127.99 (CH), 127.92 (CH), 127.90 (CH), 127.88 (CH), 127.83 (CH), 127.66 (CH), 127.52 (CH), 96.97 (CH), 82.06 (CH), 80.04 (CH), 77.74 (CH),

75.64 (CH_2), 75.08 (CH_2), 73.45 (CH_2), 73.15 (CH_2), 70.15 (CH), 68.55 (CH_2), 67.95 (CH_2), 66.52 (CH_2), 40.85 (CH_2), 29.62 (CH_2), 28.90 (CH_2), 23.35 (CH_2); HRMS (ESI) calcd for $C_{47}H_{53}NO_8Na$ ($[M + Na]^+$) 782.3669, found 782.3670.

β-4l: Colorless oil; $[\alpha]^{21}_{D}$ 12.6 (*c* 0.13, CH_2Cl_2); IR: ν 3351, 3063, 3030, 2929, 2864, 1719, 1586, 1520, 1497, 1454, 1401, 1361, 1306, 1242, 1067, 1028, 911, 824, 735, 697 cm^{-1}; ^1H NMR (500 MHz, $CDCl_3$) δ 7.37-7.22 (m, 23H, Ph-H), 7.16-7.12 (m, 2H, Ph-H), 5.06 (s, 2H, CH_2Ph), 4.91 (d, *J* = 11.10 Hz, 1H, CH_2Ph), 4.90 (d, *J* = 10.95 Hz, 1H, CH_2Ph), 4.80 (d, *J* = 10.60 Hz, 1H, CH_2Ph), 4.77 (d, *J* = 10.95 Hz, 1H, CH_2Ph), 4.71 (d, *J* = 11.10 Hz, 1H, CH_2Ph), 4.59 (d, *J* = 12.30 Hz, 1H, CH_2Ph), 4.53 (d, *J* = 12.30 Hz, 1H, CH_2Ph), 4.50 (d, *J* = 10.60 Hz, 1H, CH_2Ph), 4.36 (d, *J* = 7.85 Hz, 1H, H-1), 3.92 (q, 1H, GluOC*H*H), 3.72 (d, *J* = 10.40 Hz, 1H, H-6a), 3.66 (dd, *J* = 4.95, 10.07 Hz, 1H, H-6b), 3.62 (t, *J* = 8.70 Hz, 1H, H-3), 3.55 (t, *J* = 9.40 Hz, 1H, H-4), 3.54-3.49 (m, 1H, GluOCH*H*), 3.47-3.43 (m, 1H, H-5), 3.42 (t, *J*= 8.65 Hz, 1H, H-2), 3.13 (quintet, 2H, CH_2), 1.64 (quintet, 2H, CH_2), 1.50 (quintet, 2H, CH_2), 1.45-1.35 (m, 2H, CH_2) ppm; ^{13}C NMR (125 MHz, $CDCl_3$) δ 156.33 (C), 138.59 (C), 138.51 (C), 138.14 (C), 138.08 (C), 136.63 (C), 128.48 (CH), 128.33 (CH), 128.09 (CH), 128.04 (CH), 127.94 (CH), 127.85 (CH), 127.74 (CH), 127.58 (CH), 103.59 (CH), 84.69 (CH), 82.24 (CH), 77.90 (CH), 75.64 (CH_2), 74.97 (CH_2), 74.80 (CH), 74.72 (CH_2), 73.44 (CH_2), 69.70 (CH_2), 68.98 (CH_2), 66.54 (CH_2), 40.92 (CH_2), 29.60 (CH_2), 29.33 (CH_2), 23.27 (CH_2) ppm; HRMS (ESI) *m*/*z* calcd for $C_{47}H_{53}NO_8Na$ ($[M + Na]^+$) 782.3669, found 782.3663.

NMR Studies of Glycosylation Mechanism

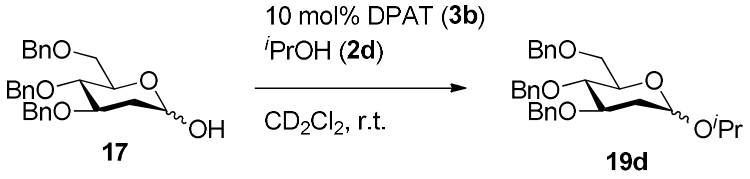

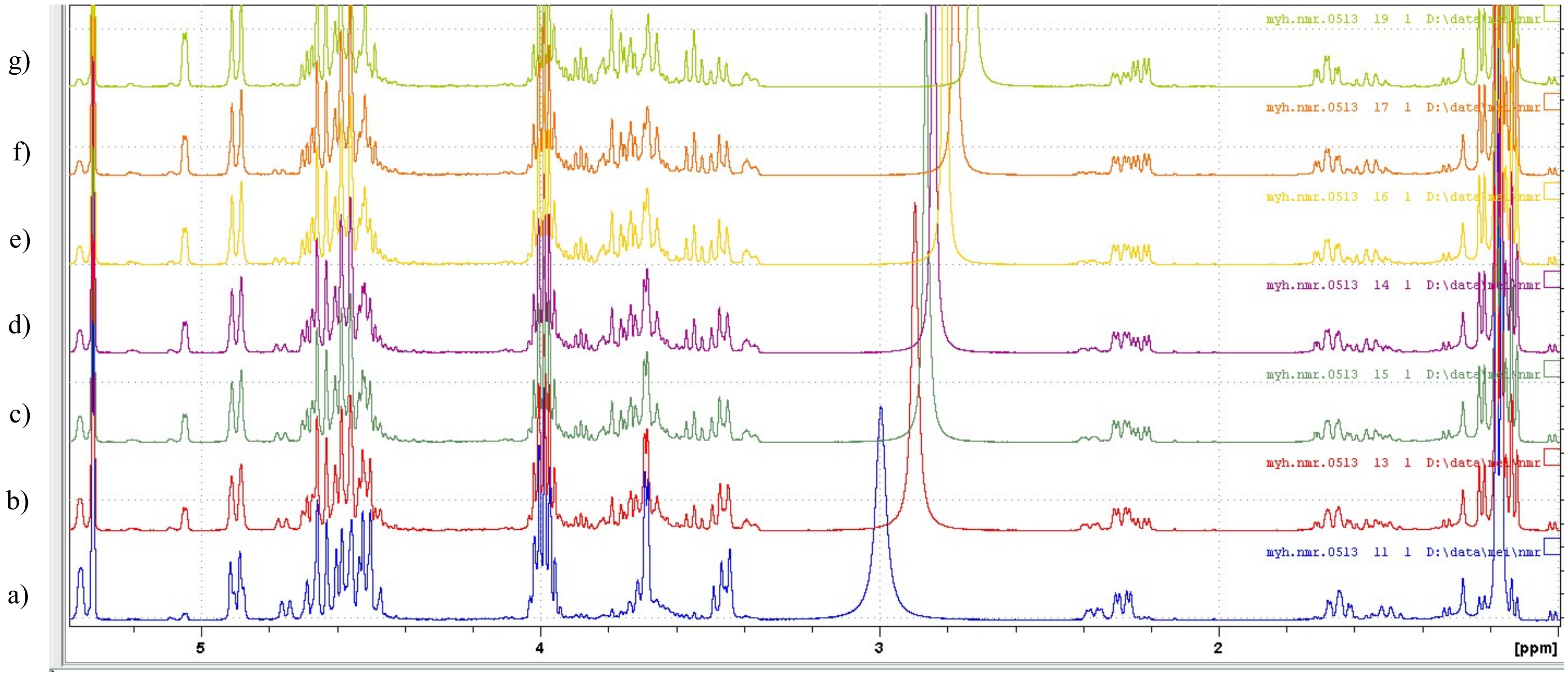

Figure S1. ^{1}H NMR spectra of the reaction of **17** and **2d** in the presence of 10 mol% of DPAT (**3b**) in CD_2Cl_2 at the ambient temperature for a) 10 min; b) 20 min; c) 30 min: d) 40 min; e) 50 min; f) 60 min; g) 120 min.

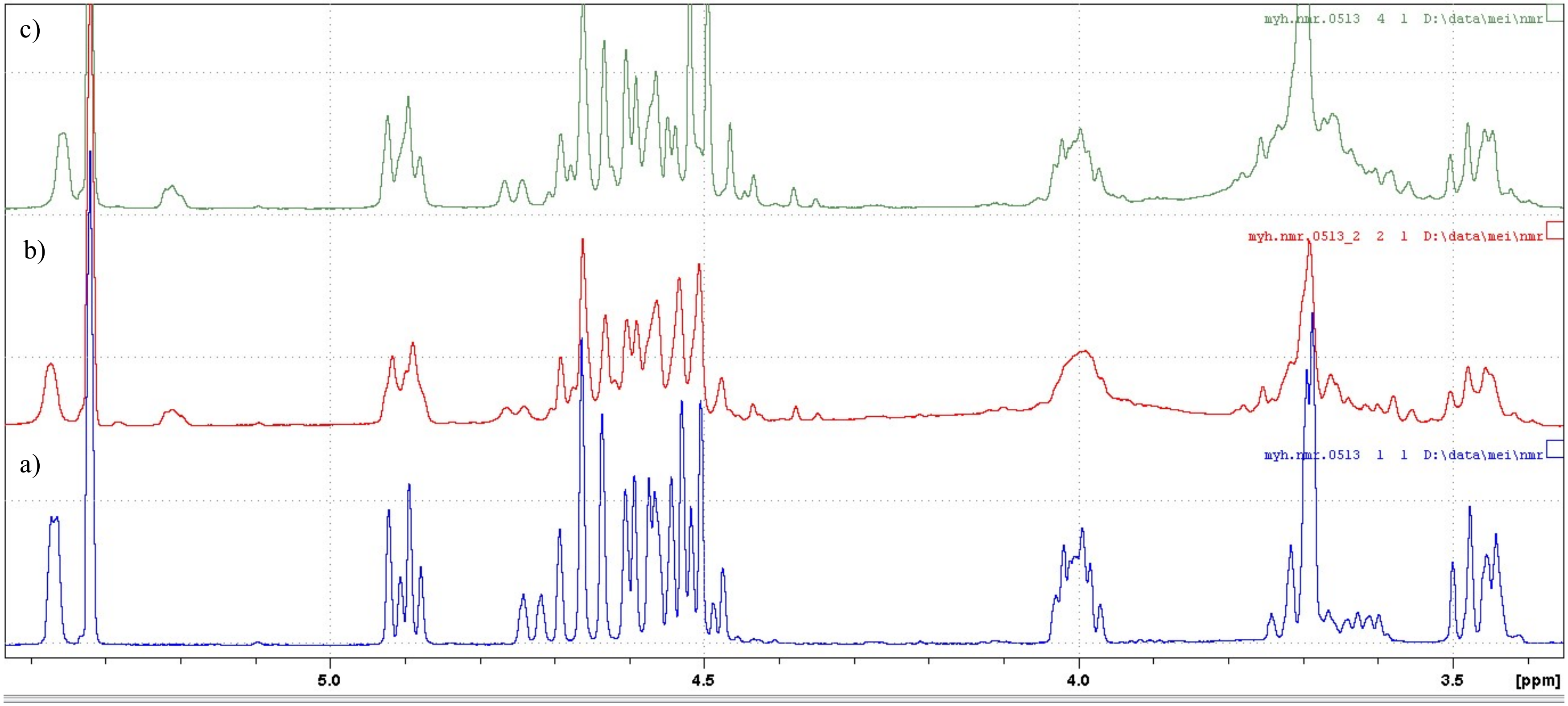

Figure S2. ^{1}H NMR spectra in CD_2Cl_2 of a) **17**; b) 10:1 of mixture of **17** and DPAT **(3b)** c) 10:1 of mixture of **17** and $[(Mes)_2NH_2][O_3S(C_6F_5)]$ (**3a**).

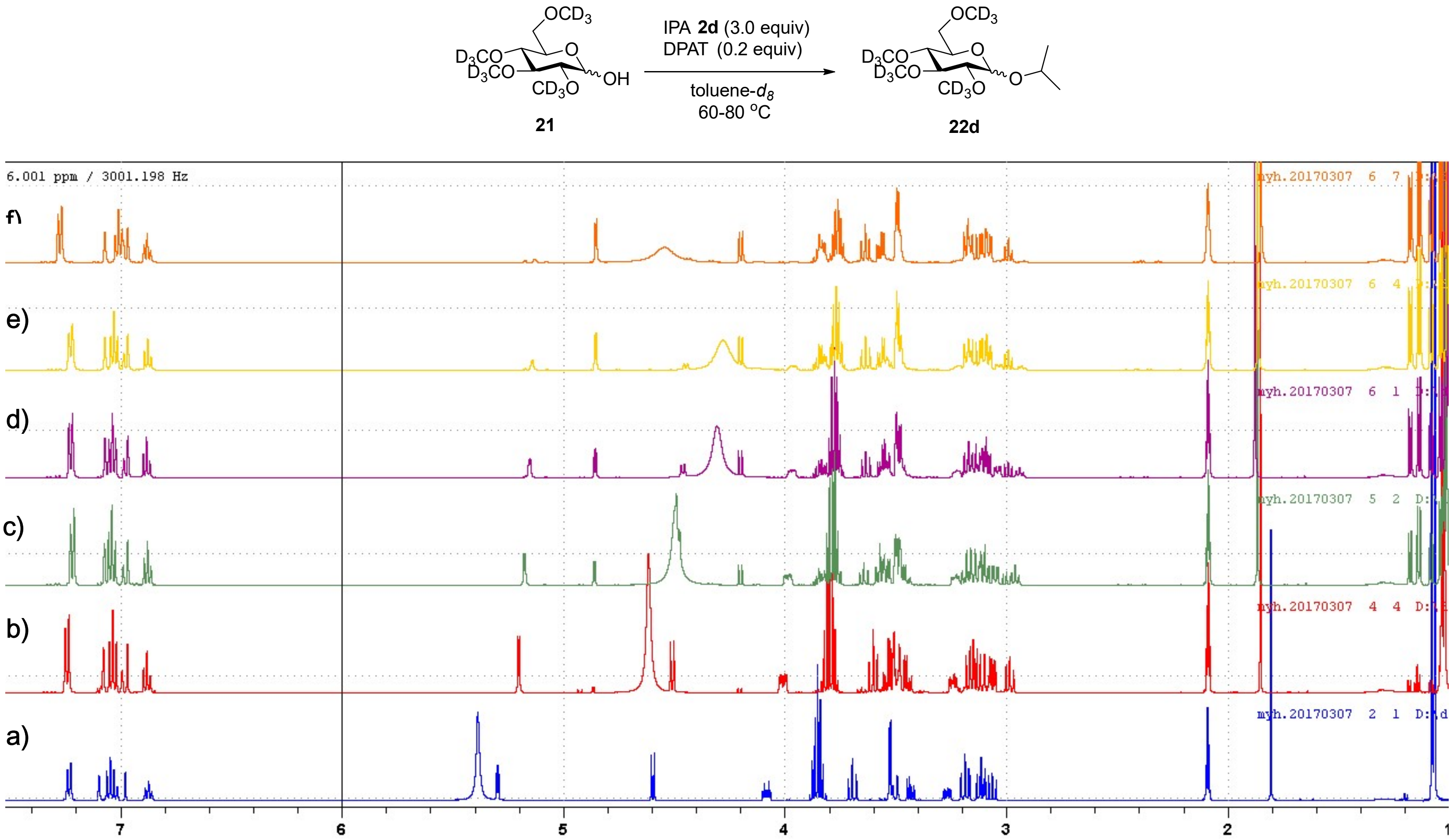

Figure S3. ^{1}H NMR spectra of DPAT-catalyzed reaction of **21**[36] and **2d** in toluene-d_8 at a) ambient temperature for 1 h; b) 60 °C for 30 min; c) 70 °C for 20 min; d) 80 °C for 10 min; e) 80 °C for 30 min; and f) 80 °C for 50 min.

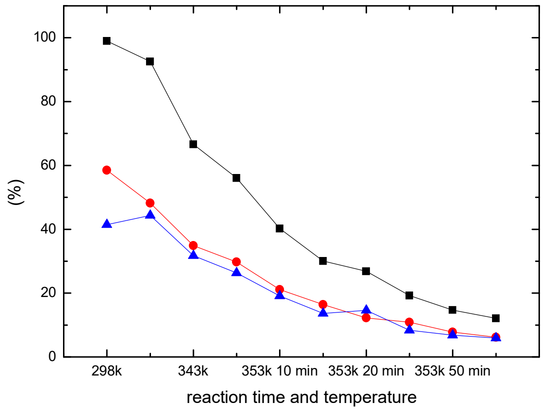

Figure S4. The normalized plots of methyl-d_3-glucopyranose **21**. Black line indicates **21**, red line indicates **21α**, and blue line indicates **21β**.

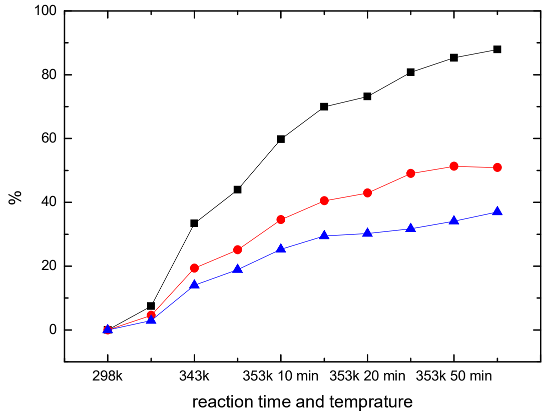

Figure S5. The normalized plots of glucoside **22d**. Black line indicates **22**, red line indicates **α-22d**, and blue line indicates **β-22d**.

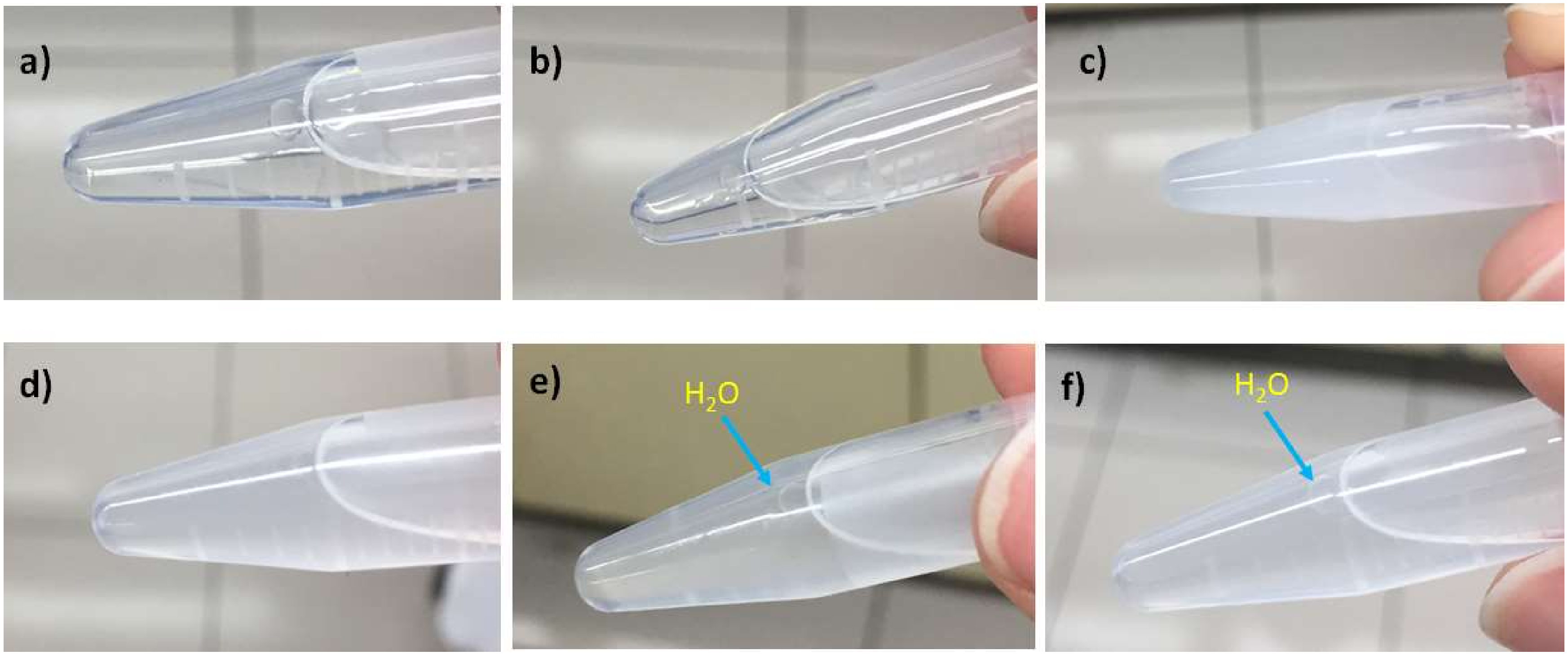

Figure S6. The water-repelling study in 1,2-dichloroethane of a) H_2O; b) 10:1 of mixture of H_2O and Ph_2NH_2; c) 10:1 of mixture of H_2O and TfOH; d) 10:1:1 of mixture of H_2O, TfOH, and succinimide; e) 10:1 of mixture of H_2O and DPAT (**3b**); f) 10:1 of mixture of H_2O and $[(Mes)_2NH_2][O_3S(C_6F_5)]$ (**3a**).

References:

(1) Dasari, B.; Jogula, S.; Borhade, R.; Balasubramanian, S.; Chandrasekar, G.; Kitambi, S. S.; Arya, P. *Org. Lett.* **2013**, *15*, 432.

(2) Tambie, M. S.; Jalsa, N. K. *J. Carbohydr. Chem.* **2015**, *34*, 545.

(3) Sudibya, H. G.; Ma, J.; Dong, X.; Ng, S.; Li, L.-J.; Liu, X.-W.; Chen, P. *Angew. Chem. Int. Ed.* **2009**, *48*, 2723.

(4) Doepner, A. M.; Aboagye, E. O.; Barrett, A. G. M. *Tetrahedron Lett.* **2015**, *56*, 3293.

(5) Motawia, M. S.; Olsen, C. E.; Denyer, K.; Smith, A. M.; Møller, B. L. *Carbohydr. Res.* **2001**, *330*, 309.

(6) Koppolu, S. R.; Niddana, R.; Balamurugan, R. *Org. Biomol. Chem.* **2015**, *13*, 5094.

(7) Bucher, C.; Gilmour, R. *Angew. Chem. Int. Ed.* **2010**, *49*, 8724.

(8) Durantie, E.; Bucher, C.; Gilmour, R. *Chem. Eur. J.* **2012**, *18*, 8208.

(9) Tatina, M.; Yousuf, S. K.; Aravinda, S.; Singh, B.; Mukherjee, D. *Carbohydr. Res.* **2013**, *381*, 142.

(10) Liao, G.; Burgula, S.; Zhou, Z.; Guo, Z. *Eur. J. Org. Chem.* **2015**, 2942.

(11) Rocheleau, S.; Pottel, J.; Huskić, I.; Moitessier, N. *Eur. J. Org. Chem.* **2017**, 646.

(12) Daragics, K.; Fügedi, P. *Tetrahedron Lett.* **2009**, *50*, 2914.

(13) Eller, S.; Collot, M.; Yin, J.; Hahm, H. S.; Seeberger, P. H. *Angew. Chem. Int. Ed.* **2013**, *52*, 5858.

(14) Lin, Y. H.; Ghosh, B.; Mong, K.-K. T. *Chem. Commun.* **2012**, *48*, 10910.

(15) Lai, Y.-C.; Luo, C.-H.; Chou, H.-C.; Yang, C.-J.; Lu, L.; Chen, C.-S. *Tetrahedron Lett.* **2016**, *57*, 2474.

(16) Geng, Y.; Kumar, A.; Faidallah, H. M.; Albar, H. A.; Mhkalid, I. A.; Schmidt, R. R. *Angew. Chem. Int. Ed.* **2013**, *52*, 10089.

(17) Kumar, A.; Kumar, V.; Dere, R. T.; Schmidt, R. R. *Org. Lett.* **2011**, *13*, 3612.

(18) Nagai, H.; Sasaki, K.; Matsumura, S.; Toshima, K. *Carbohydr. Res.* **2005**, *340*, 337.

(19) Vankayalapati, H.; Singh, G.; Tranoy, I. *Tetrahedron: Asymmetry* **2001**, *12*, 1373.

(20) Sakakura, A.; Kawajiri, K.; Ohkubo, T.; Kosugi, Y.; Ishihara, K. *J. Am. Chem. Soc.* **2007**, *129*, 14775.

(21) Levecque, P.; Gammon, D. W.; Kinfe, H. H.; Jacobs, P.; De Vos, D.; Sels, B. *Adv. Synth. Catal.* **2008**, *350*, 1557.

(22) Guchhait, G.; Misra, A. K. *Catal. Commun.* **2011**, *14*, 52.

(23) O'Brien, C.; Poláková, M.; Pitt, N.; Tosin, M.; Murphy, P. V. *Chem. Eur. J.*

2007, *13*, 902.

(24) Doores, K. J.; Davis, B. G. *Chem. Commun.* **2005**, 168.

(25) Costantino, V.; Fattorusso, E.; Imperatore, C.; Mangoni, A.; Freigang, S.; Teyton, L. *Bioorg. Med. Chem.* **2008**, *16*, 2077.

(26) van Well, R. M.; Ravindranathan Kartha, K. P.; Field, R. A. *J. Carbohydr. Chem.* **2005**, *24*, 463.

(27) Grayson, E. J.; Ward, S. J.; Hall, A. L.; Rendle, P. M.; Gamblin, D. P.; Batsanov, A. S.; Davis, B. G. *J. Org. Chem.* **2005**, *70*, 9740.

(28) Nokami, T.; Shibuya, A.; Tsuyama, H.; Suga, S.; Bowers, A. A.; Crich, D.; Yoshida, J.-i. *J. Am. Chem. Soc.* **2007**, *129*, 10922.

(29) Wang, Y.; Zhang, X.; Wang, P. *Org. Biomol. Chem.* **2010**, *8*, 4322.

(30) Das, S.; Pekel, D.; Neudörfl, J.-M.; Berkessel, A. *Angew. Chem. Int. Ed.* **2015**, *54*, 12479.

(31) Toshima, K.; Nagai, H.; Kasumi, K.-i.; Kawahara, K.; Matsumura, S. *Tetrahedron* **2004**, *60*, 5331.

(32) Verma, V. P.; Wang, C.-C. *Chem. Eur. J.* **2013**, *19*, 846.

(33) Nogueira, J. M.; Nguyen, S. H.; Bennett, C. S. *Org. Lett.* **2011**, *13*, 2814.

(34) Pachamuthu, K.; Vankar, Y. D. *J. Org. Chem.* **2001**, *66*, 7511.

(35) Balmond, E. I.; Coe, D. M.; Galan, M. C.; McGarrigle, E. M. *Angew. Chem. Int. Ed.* **2012**, *51*, 9152.

(36) Xu, G.; Moeller, K. D. Org. Lett. **2010**, *12*, 2590-2593.
